# Comparison of plant microbiota in diseased and healthy rice reveals methylobacteria as health signatures with biocontrol capabilities

**DOI:** 10.3389/fpls.2024.1468192

**Published:** 2024-10-29

**Authors:** Kakada Oeum, Malyna Suong, Kimsrong Uon, Léa Jobert, Stéphane Bellafiore, Aurore Comte, Emilie Thomas, Fidero Kuok, Lionel Moulin

**Affiliations:** ^1^ Research and Innovation Center, Institute of Technology of Cambodia, Phnom Penh, Cambodia; ^2^ Plant Health Institute of Montpellier (PHIM), IRD, CIRAD, INRAE, Institut Agro, Univ Montpellier, Montpellier, France

**Keywords:** *Oryza sativa*, plant microbiome, amplicon sequencing, bioinoculant, sustainable agriculture, plant health, phytopathogen

## Abstract

**Introduction:**

Rice (*Oryza sativa*) is a staple food worldwide, but its production is under constant pressure from both abiotic and biotic stresses, resulting in high use of agrochemicals. The plant microbiome harbours microorganisms that can benefit plant health and provide alternatives to the use of agrochemicals. The composition of plant microbiomes depends on many factors (soil composition, age, and health) and is considered a primary driver of future plant health. To identify plant microbiomes that protect against disease, we hypothesised that asymptomatic rice plants in fields under high pathogen pressure (i.e., healthy islands of plants among predominantly diseased plants) might harbour a microbiota that protects them from disease.

**Material and Methods:**

We sampled healthy and leaf-diseased plants in rice fields with high disease incidence in Cambodia and profiled their microbiota at leaf, root, and rhizosphere levels using 16S V3V4 and 18S V4 amplicon barcoding sequencing.

**Results:**

Comparison of amplicon sequence variants (ASV) of the microbiota of healthy and diseased samples revealed both disease and healthy signatures (significant enrichment or depletion at ASV/species/genus level) in both fields. The genera *Methylobacterium* and *Methylorubrum* were identified health taxa signatures with several species significantly enriched in healthy leaf samples (*Methylobacterium indicum*, *Methylobacterium komagatae*, *Methylobacterium aerolatum*, and *Methylorubrum rhodinum*). A cultivation approach on rice samples led to the isolation of bacterial strains of these two genera, which were further tested as bioinoculants on rice leaves under controlled conditions, showing for some of them a significant reduction (up to 77%) in symptoms induced by *Xanthomonas oryzae* pv. *oryzae* infection.

**Discussion:**

We validated the hypothesis that healthy plants in fields under high disease occurrence can host specific microbiota with biocontrol capacities. This strategy could help identify new microbes with biocontrol potential for sustainable rice production.

## 1 Introduction

Plants host diverse microbial communities (the plant microbiota) that are associated with the phyllosphere, root, rhizosphere, and endosphere and comprise bacteria, fungi, protists, nematodes, and viruses. These communities live in symbiosis with the plant, but their composition can shift under several conditions (high input of chemical fertilisers and pesticides, biotic or abiotic stress) leading to dysbiosis, making the plant more susceptible to stresses (Berg and Cernava, 2022).

Plant microbiota have attracted considerable interest in recent years, as harnessing plant microbiomes could help to develop more sustainable agriculture (Busby et al., 2017). Plants host a specific microbiota in their above- and below-ground parts that may play a role in their growth and tolerance to biotic and abiotic stresses (Compant et al., 2019; Trivedi et al., 2020). In the case of biotic stress, many studies have highlighted the correlation between the presence of specific taxa and disease tolerance in plants (Li et al., 2021; Liu et al., 2021, 2023; Yang et al., 2024). The isolation of such taxa and their inoculation into plants has made it possible to assess causality and identify potential microorganisms as biocontrol agents against a range of phytopathogens (Berendsen et al., 2018). Recruitment of specific microbes when plants are under pathogen attack has also been documented by altering the composition of root exudates, such as coumarins (Stringlis et al., 2019; Stassen et al., 2021). Several authors have applied the Anna Karina principle, which states that the microbiota of healthy plants is more similar, whereas diseased plants have a more diverse plant microbiota (Arnault et al., 2023). On the other hand, the composition of the pathobiota [i.e., host-associated organisms associated with reduced (or potentially reduced) health status] appears to be highly dynamic between seasons (Bartoli et al., 2018), and its specificity towards disease diversity remains poorly studied.

The initial composition of the soil microbiome has also been shown to be an indicator of future plant health (Wei et al., 2019), meaning that the variability of the soil microbiome across a field could be a main driver of disease occurrence later in plant development. Thus, the initial processes of plant microbiota assembly appear to be crucial for future plant health, and external stressors that disrupt this assembly could lead to dysbiosis (Arnault et al., 2023). Identifying the composition of healthy plant microbiomes could help develop bioinoculants to restore symbiotic microbiota and make plants less susceptible to stress. In theory, comparing the microbiota of healthy and diseased plants in agricultural fields under high pathogenic pressure should allow the detection of healthy microbiota signatures (i.e., taxa enriched in healthy plants) and disease-specific ones.

Rice (*Oryza sativa*) is a staple food for over half the world’s population and has been used as a model crop for research (Shimamoto and Kyozuka, 2002). It was one of the first model crops to be studied for its microbiome (Kim and Lee, 2020), and intensive research has been conducted to identify rice endophytes for use in sustainable agriculture (Moronta-Barrios et al., 2018; Ganie et al., 2022; Sondo et al., 2023). The main drivers of rice microbiota composition are, first, the plant compartment, followed by soil, agricultural practices, and rice genotype (Edwards et al., 2015; Zhang et al., 2022). A study on 68 *Oryza sativa* subspecies (subsp.) *indica* and 27 subsp. *japonica* cultivars showed that the main difference in root microbiota between *indica* and *japonica* subspecies were related to the presence of a nitrate importer in *indica* subspecies, linking microbiota composition to plant functional traits (Zhang et al., 2019). Rice microbiota composition also changes throughout the plant life cycle, converging in similarity during the reproductive stage (Edwards et al., 2018). Rice microbiota has also been shown to be specific compared to other crops, due to its anoxic environment during flooding (Ding et al., 2019), and the irrigation regime (irrigated versus lowland) strongly influences its composition (Barro et al., 2022). Rice is the target of many bacterial (as bacterial leaf blight, bacterial leaf streak, and phytoplasma) and fungal (as pyriculariosis, sheath rot, narrow brown spot, and brown spot) and viral (RYMV, Tungro) diseases, and phytoparasitic nematodes and insect pest (list available at http://www.knowledgebank.irri.org/). Although the use of biocontrol microbes for plant disease and pest management has been the subject of many studies (Collinge et al., 2022; Lahlali et al., 2022), few studies have compared the microbiota of healthy and diseased rice to identify health-related microbial taxa as a new source of bioinoculants for biocontrol of rice diseases.

Rice is the main crop in Cambodia, with jasmine rice being a premium rice for export. Several surveys have identified main rice pathogens: from bacterial diseases caused by *Xanthomonas oryzae* pv. *oryzae* (*Xoo*, bacterial leaf blight) and pv. *oryzicola* (*Xoc*, bacterial leaf streak), *Acidovorax avenae* subsp. *avenae*, *Burkholderia gladioli*, *Pseudomonas fuscovaginae*, *B. cepacia*, and *Pantoea ananatis* (Cother et al., 2010; Socheath et al., 2023), to orange leaf phytoplasma (ROLP) (Ong et al., 2021), to rice fungal diseases as rice blast (*Pyricularia oryzae*), narrow brown spot (*Sphaerulina oryzinae/Cercospora janseana*), brown spot (*Bipolaris oryzae*), and rice brown leaf spot (*Curvularia lunata*) (Fukuta et al., 2014; Tann and Soytong, 2016), or phytoparasitic nematodes (Suong et al., 2019; Masson et al., 2022).

In this study, we tested the hypothesis that healthy plants in fields with high disease incidence (i.e., healthy plants in rice fields among diseased plants) would host a protective microbiota. We identified rice fields in Cambodia under high multi-pathogen pressure in order to compare the microbiota of diseased (leaf disease) and healthy rice, looking for health signature microbial taxa. We analysed leaf, root, and rhizosphere microbiota (using 16S and 18S barcode amplicon sequencing) in diseased and healthy rice plants in two fields. Health signature taxa were detected, and a cultivable approach was developed to isolate the signature taxa and test them as bioprotective agents against several rice pathogens.

## 2 Materials and methods

### 2.1 Sampling of rice material

Two rice fields showing high foliar disease incidence (number of plants showing leaf symptoms > 70%) were sampled in Cambodia in November 2021. Map and characteristics of each site are detailed in **Supplementary Table S1**. The first field was close to Rovieng village in Preah Vihear province (GPS coordinates: lat. 13.416694, long. 105.14509), under conservation agriculture practice [with no-till, no chemical inputs, organic fertilizer (biochar 2 tons/ha), and cover crops between rice cycle]. The rice variety was Phka Rumduol (fragrant rice, long cycle). The second field was located in Preak Sdei (gps coordinates: lat. 11.05584, long. 105.04581) and managed with conventional farming (tillage, high chemical inputs), with a local indica rice variety. Sampling, analysis, and exportation of material were authorised by the Cambodian Ministry of Environment permit no. 036 MoE. On each field, roots, rhizosphere, and leaves were harvested on 20 plants ranging from no leaf symptoms (10 plants) to almost full leaf symptoms (10 plants; symptoms covering 50%–90% of leaf). Fields were harvested at the ripening stage, which is the stage where leaf symptoms are the most visible on rice. Samples were conserved at 4°C for 24 h before being processed for DNA extraction.

### 2.2 DNA extraction

Rice leaves and roots were homogenised in liquid nitrogen in a sterile mortar, and 250 mg of powder was transferred to PowerBead tubes (Qiagen, Promega, USA). Extraction was then performed according to the protocol provided by the supplier. For rhizosphere samples, 250 mg of soil was used for DNA extraction. All samples were extracted using the DNeasy PowerSoil Pro Kit (Qiagen, Promega, USA), following the protocol provided with the kit. A control with no sample was included to remove any contaminant from the kit after sequence data production. After extraction, DNA concentration, and quality were assessed on a Nanodrop spectrophotometer.

### 2.3 Amplicon libraries and sequencing

Quality control of DNA, PCR amplification, library construction, and MiSeq Illumina sequencing were performed by Macrogen (Seoul, South Korea). For amplicons, primers 16S_337F (5′-CCTACGGGNGGCWGCAG-3′) and 16S-805R (5′-GACTACHVGGGTATCTAATCC-3′) (Sinclair et al., 2015), and V4F (5′-CCAGCAGCCGCGGTAATTCC-3′) and V4R (5′-ACTTTCGTTCTTGATTAA-3′) (Choi and Park, 2020), were used to amplify the V3–V4 region of the 16S rDNA gene and the V4 region of the 18S rDNA gene, respectively. The kit used for library construction was the Herculase II Fusion DNA Polymerase Nextera XT Index V2 kit. Libraries were sequenced on an Illumina Miseq, with 2 × 300 bp reads (paired-end). The number of sequences varied from 35,000 to 86,000 (mean at 56,715) for 16S libraries and from 33,000 to 82,000 (mean at 53,091) for 18S libraries. The amplicon sequencing data (fastq) for this study are accessible in the ENA (European Nucleotide Archive, https://www.ebi.ac.uk/ena) database under the Bioproject PRJEB70289 (ERP155223).

### 2.4 Microbiota analyses

16S and 18S raw amplicon barcoding data were demultiplexed and processed using the Bioconductor Workflow for Microbiome Data analysis (Callahan et al., 2016b), which is based on DADA2 (Callahan et al., 2016a) that infers amplicon sequence variants (ASV) from raw sequence reads. Forward and reverse reads were trimmed on 5′ (trimleft) to remove primers and adapters, and then quality-truncated at 280 bp and 205 bp, after checking quality (using the plotQualityProfile function in dada2). The dada2 denoise-paired function with default parameters was used to correct sequencing errors and infer exact ASVs. Then, forward and reverse corrected reads were merged with a minimum 20 bp overlap, and the removeBimeraDenovo function from DADA2 was used to remove chimeric sequences. The numbers of reads filtered, merged, and non-chimeric are indicated in **Supplementary Table S2**. For 16S sequences, a mean of 51% of reads passed all filters (denoising, merging, non-chimeric), with a minimum of 5,561 and a maximum of 27,165 reads in filtered libraries. For 18S sequences, a mean of 86% of reads passed all filters, with a minimum of 12,106 and a maximum of 34,047 reads.

ASV were then assigned at the taxonomic level using the DADA2 AssignTaxonomy function, with the Silva reference database (silva_nr_v138_train_set), and species were added with the addSpecies function (database silva_species_assignment_v138) (Quast et al., 2013). We subsequently filtered out plants (mitochondria and chloroplast) from 16S to keep only ASV assigned to the Bacteria or Archaea kingdoms in 16S ASV and 18S plant reads to keep only eukaryotic fungi and microfauna for 18S ASV. Final filtering was performed to remove ASV with <10 reads across all libraries and ASV from the negative control. A dataset of 10,530 ASV for 16S and 3596 ASV for 18S was used for subsequent diversity analyses. Taxonomic affiliation was refined for the Nematoda phylum by aligning ASV 18S sequences to a refined set of 18S sequences from reference isolates (Helder et al., 2014) and enriched by us with rice phytoparasitic nematodes (*Hirschmanniella* and *Meloidogyne* species). Sequences and ASV were aligned, and a phylogenetic tree was built using MEGA11 (Tamura et al., 2021) (tree available as **Supplementary Figure S1**). Metadata and ASV tables were uploaded to the NAMCO server for downstream microbiota diversity analyses (https://exbio.wzw.tum.de/namco/) (Dietrich et al., 2022). NAMCO is a microbiome explorer server based on a set of R packages (including Phyloseq). Alpha-diversity analyses (observed richness, Shannon and Simpson diversity, and subgroup pairwise Wilcoxon test) were performed with Phyloseq and tidyverse, ggpubr, rstatix, and multcompView R packages and plotted with ggplot2. Beta-diversity (NMDS, PERMANOVA) was performed with Phyloseq and Vegan. Data were normalised with the centre-log ratio method, and a Wilcoxon (α=0.05) test with Bonferroni correction was performed across conditions (healthy versus disease) with the ALDEx2 package (Fernandes et al., 2014). Quantification of enriched or depleted taxa (on ASV > 50 reads across all libraries for each organ and filed) was visualised on heat trees using metacoder, an R package for graphing publication-ready plots of hierarchical data that includes quantitative representation of up to four arbitrary statistics simultaneously in a tree format by mapping statistics to the colour and size of tree nodes and edges (Foster et al., 2017). R scripts for the DADA2 pipeline and Metacoder heat trees are available on GitHub at https://github.com/lmoulin34/Oeum_RiceMicrobiomeHealthSignatures.

### 2.5 PCR detection of pathogens

We run a PCR-based pathogen diagnostic using PCR primers described in the literature as specific for the main diseases (listed in **Table 1** with PCR cycles and references). PCR were done using the same leaf DNAs extractions as the ones used for amplicon barcoding. The investigated pathogen species were *Bipolaris* spp. (brown spot), *Xanthomonas oryzae* pv. *oryzae* [bacterial leaf blight (BLB)] and *X. oryzae* pv. *oryzicola* [bacterial leaf streak (BLS)], *Pyricularia oryzae* (blast), *Cercospora* spp. (narrow Brown spot), *Burkholderia glumae* (panicle blight), *Pantoea ananatis* (leaf blight), and the rice orange leaf phytoplasma (ROLP). For narrow brown spot, as this disease was abundant in our samples and there were no specific primers in the literature, we designed a new pair of specific primers based on an alignment of the gadph gene, that amplifies *Cercospora* spp. (**Table 1**).

**Table 1:** Primers used for phytopathogen specific detection by PCR in DNA samples.

Disease/pathogen	Target gene	Primer name	Primer sequence (5′–3′)	Size (bp)	PCR cycling	Reference
Brown spot (*Bipolaris* spp.)	gadpH	GAPDH_Bspf GAPDH_R	AGCACTCCCTCAACCCRGAA TTCTCGGTGGTGGTGAAGAC	296	2 min 96°C; then 35 cycles: 96°C 1 min, 55°C 1 min, 72°C 50 s; final, extension 10 min 72°C	(Kabore, 2022)
Blast (*Pyricularia oryzae*)	Pot2 transposon	pfh2a pfh2b	CGTCACACGTTCTTCAACC CGTTTCACGCTTCTCCG	687	3 min 95°C; then 35cycles: 95°C 30 s, 55°C 30 s, 72°C 1 min 30 s; final extension, 7 min 72°C	(Harmon et al., 2003; Paul et al., 2022)
BLB and BLS (*Xanthomonas oryzae* pv *oryzae* and pv *oryzicola*)	Hyp. protein	Xo3756F Xo3756R	CATCGTTAGGACTGCCAGAAG GTGAGAACCACCGCCATCT	331	2 min 95 °C; then 30 cycles: (94°C 30 s, 55°C 30 sec, 72°C 30 s); final extension, 7 min 72°C	(Lang et al., 2010)
Narrow Brown leaf spot (NBLS) (*Cercospora*)	gdph	CercoGpdFor CercoGpdRev	TGTCTTYACCACYACCGA TGGSACACGCATRGACAT	411	2 min 95°C; then 35 cycles: 95°C 45 sec, 55°C 45 sec, 72°C 45 s; final extension, 10 min 72°C	This study
Panicle blight (*Burkholderia glumae*)	toxB	toxB-F toxB-R	GCATTTGAAACCGAGATGGT TCGCATGCAGATAACCRAAG	508	2 min 96 °C; then 30 cycles: 94°C 30 s, 58°C 30 sec, 72°C 45 s; final extension, 7 min 72°C	(Bangratz et al., 2020)
Bacterial blight (*Pantoea* spp.)	atpD	PANKK262F PANKK263R	GCGAGCCAATCGACATTA CGAGTAACCTGAGTGTTCAG	263	2 min 96 °C; then 30 cycles: 94°C 30 s, 58°C 30 sec, 72°C 45 s; final extension, 7 min 72°C	(Bangratz et al., 2020)
ROLP (rice orange leaf phytoplasma)	16SrDNA	P1 P7 R16F2n R16R2	AAGAGTTTGATCCTGGCTCAGGATT CGTCCTTCATCGGCTCTT GAAACGACTGCTAAGACTGG TGACGGGCGGTGTGTACAAACCCCG	1,900 1,200	Nested PCR: P1/P7: 94°C for 2 min; 30 cycles of 94°C for 30 s, 52°C for 30 s, and 72°C for 2 min; final 72°C for 10 min. Second PCR with R16F2n R16R2: same cycling annealing T° 50°C.	(Li et al., 2015)

### 2.6 Culturable approach of *Methylobacterium* and *Methylorubrum* strains

Plant samples (conserved at 4°C) were crushed in a sterile mortar, resuspended in sterile water, and 50 µl of serial dilutions of the suspension was plated on M72 medium [per L: K_2_HPO_4_, 1.2 g; KH_2_PO_4_, 0.62 g; CaCl_2_.6H_2_O, 0.05 g; MgSO_4_.7H_2_O, 0.2 g; NaCl, 0.1 g; FeCl_3_.6H_2_O, 1 mg; (NH_4_)2SO_4_, 0.5 g; and trace elements, 1 ml (per L: CuSO_4_.5H_2_O, 5 mg; MnSO_4_.H_2_O, 7 mg; Na_2_MoO_4_.2H_2_O, 10 mg; H_3_BO_3_, 10 mg; ZnSO_4_.7H_2_O, 70 mg; CoCl_2_.6H_2_O, 5 mg; pH adjusted to 7.0; agar, 15 g L^−1^] supplemented with 0.5 ml of methanol per 100 ml of medium (methanol filtered at 0.22 µm), added after autoclaving of media (20 min, 120°C). To avoid fungal growth, cycloheximide at 10 µg ml^−1^ final concentration was added (addition after medium sterilisation; cycloheximide was filtered at 0.22 µm). Petri dishes were placed in an incubator at 28°C until apparition of isolated colonies. Pink pigmented single colonies were purified on Tryptic Soy Agar medium (TSA, Sigma-Aldrich, France) two times, then inoculated to 20 ml of Tryptic Soy Broth medium (TSB, Sigma-Aldrich, France), and after, growth cultures were supplemented with sterile glycerol (20% final concentration) and frozen at −80°C. A 16S rDNA fragment of each colony was amplified (approximately 1,500 bp with FGPS6/FGP1509 primers as described in Sondo et al. (2023) and sequenced at Genoscreen company (Sanger method) using the 16S-1080r internal primer. Partial 16S rRNA sequences (approximately 1,000 bp) of four strains were submitted to GenBank under number PP724422 to PP724426.

### 2.7 Phylogenetic analysis

Phylogenetic analyses were conducted in MEGA11 (Tamura et al., 2021). A phylogenetic analysis was performed to compare the 16SrDNA V3V4 partial sequence of ASV, cultured strains, and closely related type strains of species belonging to *Methylobacterium* and *Methylorubrum* genera. The phylogeny was inferred by using the Maximum Likelihood (ML) method and Kimura two-parameter model, a gamma distribution of sites (five categories), and 1,000 bootstrap replicates. Initial tree(s) for the ML heuristic search were obtained automatically by neighbour joining. All positions with <95% site coverage were eliminated (partial deletion option), resulting in a total of 400 positions in the final dataset.

### 2.8 Plant biocontrol tests

The Phka rumduol variety (Cambodian jasmine rice) was used for the plant biocontrol test experiments. Seeds were sowed on individual pots filled with potting soil (GO M2, Jiffy, composed of 65% peat, 20% coir, 10% perlite, 5% sand, pH 5) and grown in a greenhouse (12 h day/night, 90% humidity). *Methylobacterium* and *Methylorubrum* strains (used for leaf spraying to assess their bioprotective capacities against a pathogen infection) and the phytopathogenic *Xanthomonas oryzae* pv. *oryzae* (Xoo) are listed in **Table 2**. *Xanthomonas Xoo* strains CIX4551 were isolated on Peptone Sucrose Agar (PSA) medium from Sen Krao Ob leaf symptoms sampled in Battambang province and characterised by multiplex PCR. Its virulence was assessed prior to our biocontrol testing on Phka rumduol variety.

**Table 2:** List of strains used in this study.

Strain name	Genus/species	Plant organ	Location	Reference
Leaf-sprayed bacteria used in biocontrol tests				
ABIP 3562	*Methylobacterium* sp. (close to *M. oryzae*)	Leaf	Preak Sdei	this study
ABIP 3533	*Methylobacterium* sp. (close to *M. terrae*)	Root	Preak Sdei	this study
ABIP 3560	*Methylorubrum* sp. (close to *Mr. rhodinum*)	Leaf	Preak Sdei	this study
ABIP 3561	*Methylorubrum* sp. (close to *Mr. rhodinum*)	Leaf	Preak Sdei	this study
*Xanthomonas* strains (bacterial leaf blight)				
CIX4551	*X. oryzae* pv. *oryzae*	Leaf	Battambang	this study

*Methylobacterium* and *Methylorubrum* strains were plated from glycerol stock on TSA50% plates for 4 days, then three colonies were inoculated to 20 *ml* broth TSB and grown for 24 h. Cultures were centrifuged for 10 min at 4,000 rpm, the pellet was resuspended in sterile water at OD=0.1, and 0.5 ml of the mixture was sprayed by plant using mini hand sprayers, on the leaves of rice (Phka rumduol) grown on pot soil. For each strain of *Methylobacterium* and *Methylorubrum*, we inoculated 10 plants (individual pots) at 2 weeks of growth, and 10 more plants were used as control by spraying sterile water on their leaves. Pots were randomised every 2 days. At 4 weeks of growth, rice leaves were infected with *Xanthomonas oryzae* pv. *oryzae (Xoo)* by leaf clipping. To prepare the pathogen inoculum, the *Xoo* CIX4551 strain was plated from glycerol stock at minus 80°C on PSA Petri dish for 2 days at 28°C, then resuspended in sterile water and diluted to obtain a culture at OD600 nm= 0.2. For leaf clipping, a scissor was dipped into *Xoo* culture and used to cut the tips of rice leaves to remove a length of approximately 2 cm. As a negative control, leaves were clipped with a scissor dipped in sterile water to assess wound spread. Symptoms size (from the cutting point) was measured 2 weeks after infection.

### 2.9 Plant tests statistics

Phenotypic data from the biocontrol test were analysed on R studio (R version 4.3.1) using the packages rstatix, multcompView, ggpubr, and ggplot2. Global mean differences across conditions were assessed with Kruskal–Wallis test (α=5%), while Wilcoxon pairwise tests (α=5%) were performed between conditions. The R script used is available at https://github.com/lmoulin34/Oeum_RiceMicrobiomeHealthSignatures.

## 3 Results

### 3.1 Alpha and beta diversity of 16S and 18S amplicon sequences of root, rhizosphere, and leaf samples from diseased and healthy rice

**Figure 1:**
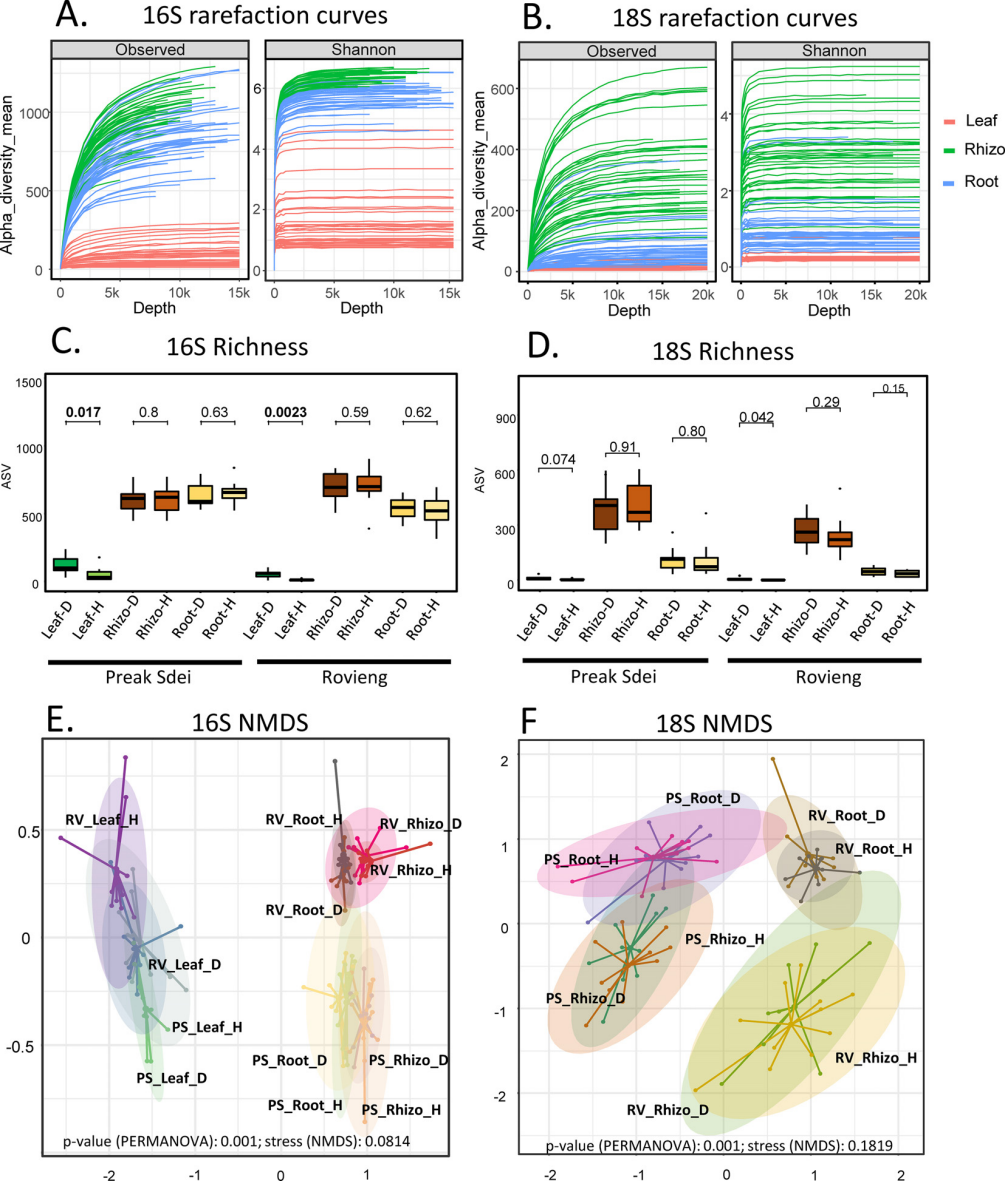
Rarefaction curves of 16s and 18S amplicon sequences **(A, B)**, richness (observed and Shannon) **(C, D)**, and NMDS of beta-diversity **(E, F)**. Rhizo, rhizosphere; PS, Preak Sdei; RV, Rovieng; H, healthy; D, disease.

A total of 40 individual rice plants (showing either leaf symptoms or no leaf symptoms; 20 per field, in two fields) were analysed in this study, on three compartments (leaf, root, and rhizosphere). The two fields differ in terms of soils and geography (separated by 300 km), agricultural practice (conservation agriculture versus conventional agriculture), and rice variety (Phka rumduol versus local indica rice), as detailed in **Supplementary Table S1**. After DNA extraction (see *Material and methods*), DNAs were analysed by amplicon barcoding and sequencing with two barcodes (16S V3V4 and 18S V4 fragments), totalising 240 libraries. Reads quality and filtering steps are given in *Material and methods* and in **Supplementary Table S2**. A final dataset of 10,530 ASV for 16S and 3596 ASV for 18S was used for alpha and beta diversity analyses. The ASV count tables for 16S and 18S with their sequence, taxonomic affiliation, and metadata are available in **Supplementary Table S3**. The rarefaction curves (**Figure 1A** for 16S and **Figure 1B** for 18S) showed that the sequencing depth was sufficient to capture the diversity of bacteria and microeukaryotes in the leaves, roots, and rhizosphere. The richness (**Figure 1C** for 16S and **Figure 1D** for 18S) was lower in the leaves compared to the root or rhizosphere. Richness for 18S reads in the leaves was very low, and several samples contain only plant reads; the leaf 18S dataset was thus not exploited further. This problem did not occur in the root and rhizosphere 18S libraries and is probably due to the low diversity of microeukaryotes on leaves. Richness of 16S sequences in the leaves was significantly higher in the diseased plants compared to healthy ones (**Figure 1C**). The beta diversity of 16S and 18S sequences for each sample was represented by NMDS and is shown in **Figures 1E, F**, respectively. A PERMANOVA test on beta diversity indices showed significant differences across the leaf, root, and rhizosphere, and the two sampled fields. PERMANOVA tests were also performed on reduced datasets to compare healthy and diseased samples for each organ in each field. Only leaves showed a significant difference between healthy and diseased samples in the PERMANOVA test in the 16S dataset (Preak Sdei p = 0.001; Rovieng p = 0.013; **Supplementary Figure S2**).

### 3.2 Detection of foliar pathogens in amplicon sequences and validation by specific PCR

**Figure 2:**
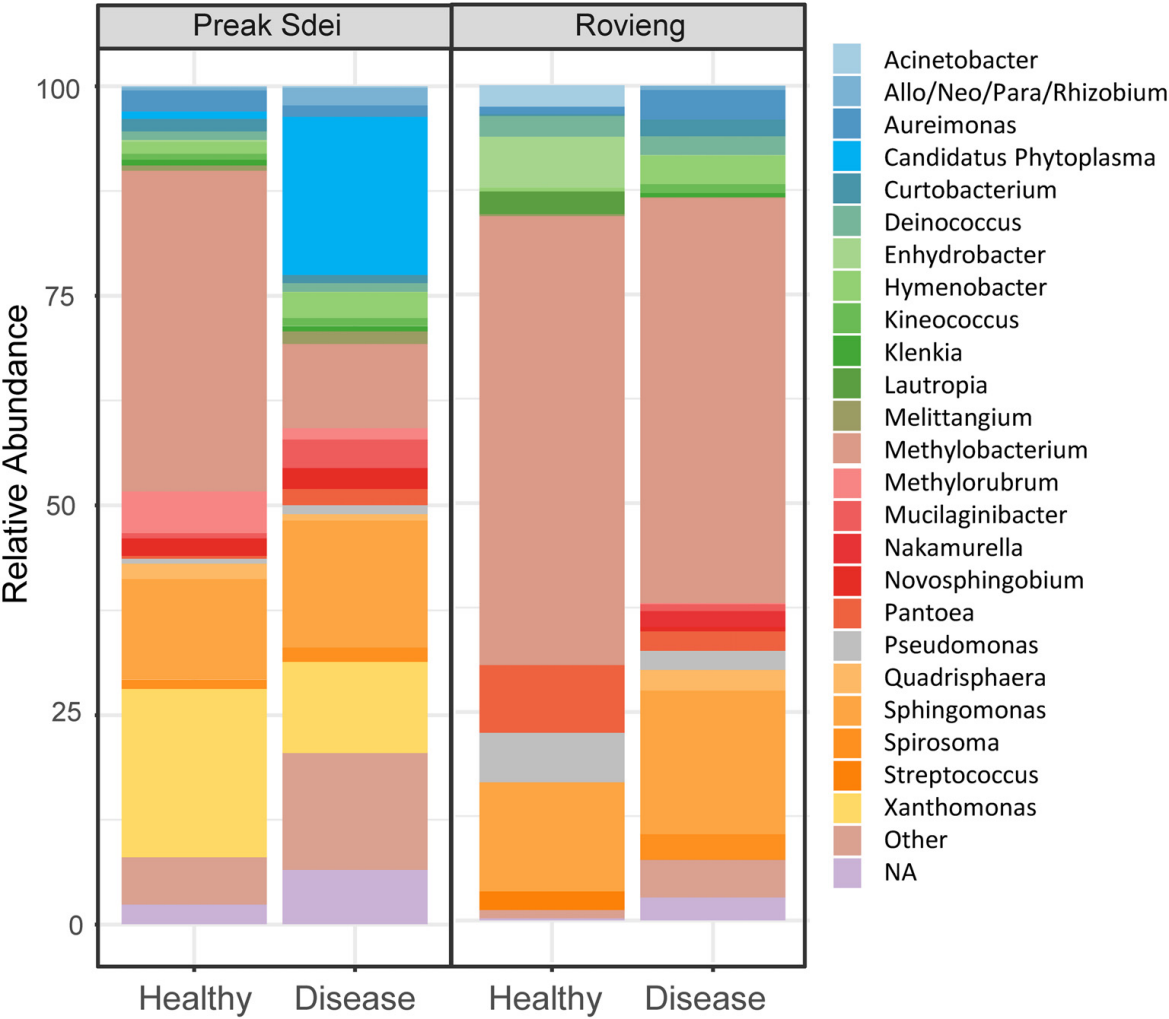
Taxonomic binning at genus level of the top 25 most abundant 16S ASV in the leaf samples in Preak Sdei and Rovieng. Binning was made on relative abundance of genera, on normalised data (centre-log ratio).

In order to detect which pathogens were present in the leaves of our rice samples from each field, we searched for pathogens in the 16S and 18S ASV sequence datasets. We show in **Figure 2** the taxonomic binning of top 25 genus (by relative abundance) detected in the 16S amplicon sequences of leaves. We detected several bacterial genera known to contain rice pathogens, as *Xanthomonas*, *Phytoplasma*, *Pantoea*, or *Sphingomonas*. As the 18S amplicon sequences contain mainly rice reads, we could not detect pathogens in the sequence dataset. In order to detect the presence of fungal pathogens and also to confirm the presence of bacterial ones (identified in the 16S ASV data), we used a PCR diagnostic with primers from the literature that amplify specific genes from phytopathogens of the main rice diseases (see *Material and methods* and **Table 1** for primer list). We searched for *Xanthomonas oryzae* pathogens (both pathovars *oryzae* and *oryzicola*), *Sphaerulina* and *Cercospora* spp. (narrow brown spot), *Pyricularia oryzae* (blast), *Bipolaris* spp. (brown spot), rice orange leaf phytoplasma (ROLP), *Burkholderia glumae* (panicle blight), and *Pantoea ananatis* (leaf bacteriosis) pathogenic species. PCR amplification on leaf DNA (same DNA as used for amplicon barcoding) was positive for several pathogens. PCR pathogen detection results are presented in **Table 3**. We sent several amplicon bands for sequencing (Sanger method) to evaluate the new primers designed for narrow brown spot detection, and the detected phytoplasma, and Blast analysis revealed that these amplicon sequences belong to the targeted phytopathogens (see **Supplementary Table S4**). We confirmed the presence of several pathogens in the diseased leaves. ROLP and narrow brown spot were the most common in Preak Sdei and Rovieng samples, respectively. *Xanthomonas oryzae* was also detected in Preak Sdei (five samples) and in Rovieng (one sample), while we did not detect any phytopathogenic *Pantoea*, *Bipolaris* spp., or *Burkholderia glumae*.

**Table 3:** Specific PCR amplification of rice phytopathogens on DNA samples from Preak Sdei and Rovieng symptomatic leaves.

Phytopathogen	Preak Sdei	Rovieng
*Xanthomonas oryzae* (*Xoo* + *Xoc*)	5/10	1/10
*Sphaerulina* and *Cercospora* spp. (narrow brown spot)	3/10	10/10
Rice orange leaf phytoplasma (ROLP)	9/10	3/10
*Bipolaris oryzae* (brown spot)	0/10	0/10
*Burkholderia glumae* (panicle blight)	0/10	0/10
*Pantoea* spp. (leaf blight)	0/10	0/10

Primers used and their references are listed in **Table 1**. A total of 10 leaves were tested by field. Numbers indicate the number of positive PCR products obtained of the correct size among the 10 leaves tested.

### 3.3 Differential analysis of leaf microbiota between leaf-diseased and healthy rice

**Figure 3:**
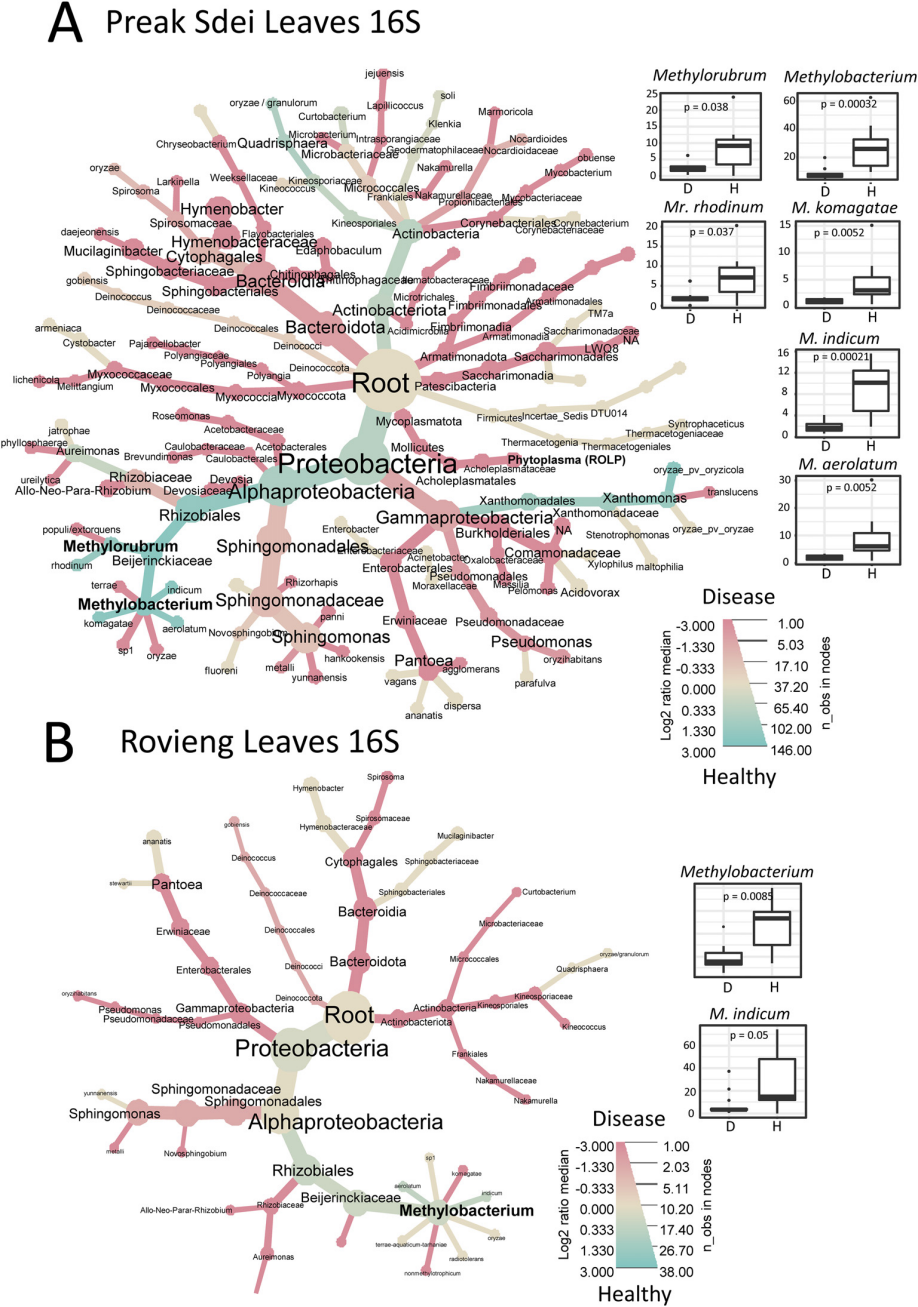
Heat trees representing differentially enriched taxa in Preak Sdei **(A)** and Rovieng **(B)** leaf samples. Enriched taxa in healthy or diseased samples are coloured in blue and red according to log2 fold proportions between the two categories of samples, respectively. ASV were filtered at 50 minimum total abundance in leaf samples. Node size is according to numbers of observed taxa counts. Box plots on right size shows genera and species detected as enriched significantly in healthy samples (p-value is a Wilcoxon test (α= 0.05) with Bonferroni correction); D stands for diseased sample and H for healthy sample.

We analysed our leaf microbiota datasets for differential enrichment between diseased and healthy leaf samples, for each field. We compared the relative abundance of each ASV in healthy and diseased samples by visualisation in a heat tree of log2 fold enrichment using metacoder (see *Material and methods*). The trees in **Figure 3** show the distribution of 16S leaf ASV at different taxa levels, according to their abundance (node size) and enrichment (blue for enrichment in healthy samples and red for enrichment in diseased samples). In parallel, we performed Wilcoxon tests (α = 0.05, with Bonferroni multiple test correction method) on centre-log ratio normalised abundances to identify bacterial taxa (at genus and species level) with significant abundance differences in healthy and diseased samples. We present all the significantly enriched taxa (either in diseased or healthy samples) in **Supplementary Figure S3A** (Preak Sdei leaf) and **Supplementary Figure S3B** (Rovieng leaf) and show only the enriched taxa in healthy leaves in **Figure 3**.

In the 16S differential analysis of Preak Sdei leaves (**Figure 3A**), we observed an enrichment of the genera *Methylobacterium* and *Methylorubrum* in healthy samples (Wilcoxon test, p=0.00032 and p=0.038, respectively). Differential analysis at the species level (on relative abundances) revealed that three *Methylobacterium* species (*M. komagatae*, *M. indicum*, and *M. aerolatum*) were enriched (p<5%), box plots in **Figure 3A**), while only one *Methylorubrum* species differed significantly (*Mr. rhodinum*). The heat tree also shows a log2 ratio enrichment of two other species in healthy leaves, *Quadrisphaera oryzae* an*d Xanthomonas oryzae*, but these were not significantly different in Wilcoxon tests. In diseased samples, 31 species were detected as enriched, including *Pantoea* spp., *Sphingomonas* spp., and *Xanthomonas* sp. (**Supplementary Figure S3A**).

In the 16S differential analysis of Rovieng leaves (**Figure 3B**), we also found the genus *Methylobacterium* enriched in healthy samples (Wilcoxon test, p = 0.0085), including the species *M. indicum* (Wilcoxon test, p=0.05). A total of 14 species were enriched in diseased samples, including *Curtobacterium* sp., *Hymenobacter* sp., *Klenkia* sp., *Nakamurella* sp., and *Spirosoma* sp.

### 3.4 Taxonomic binning of root and rhizosphere ASV

**Figure 4:**
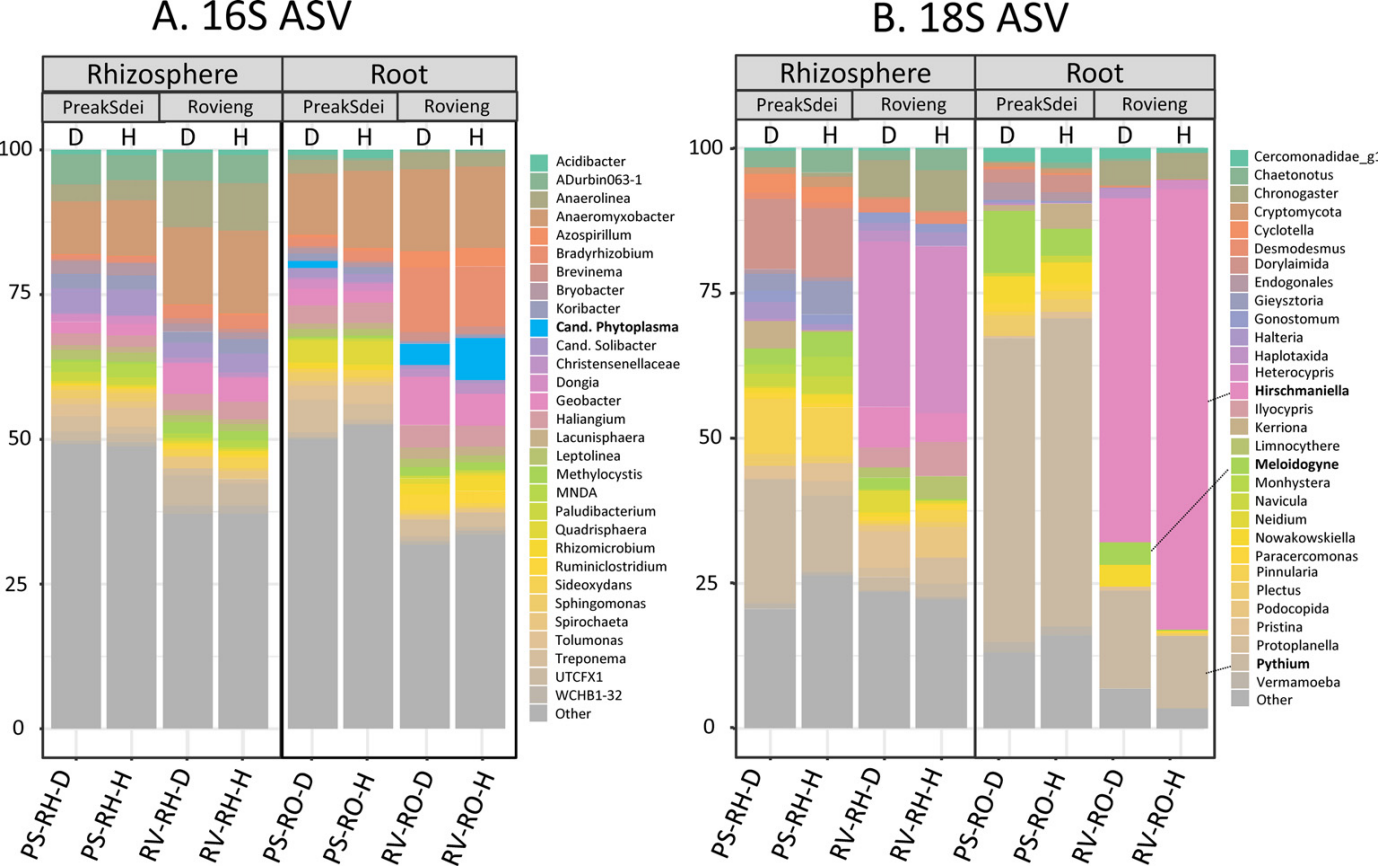
Taxonomic binning of 16S **(A)** and 18S **(B)** ASV genera (top 30) in root and rhizosphere. Binning was made according to compartment [rhizosphere (RH) or root (RO)], field (PS, Preak Sdei; RV, Rovieng) and health status of rice (D, leaf-diseased; H, healthy leaf). Binning was made on relative abundance of taxa, on normalised data (centre-log ratio). Genera in bold are known rice root phytopathogens and pest.

We analysed the taxonomic distribution of ASV in the 16S and 18S amplicon datasets in each compartment and each field and according to the health status of the samples. In **Figure 4**, we present the taxonomic binning (top 30) at the genus level of the relative abundances of 16S (**Figure 4A**) and 18S (**Figure 4B**) ASV. We have also plotted the 16S and 18S diversity of (top 30 genera) for all root and rhizosphere samples in **Supplementary Figure S4**. In the 16S ASV dataset, we observed the presence of the genus *Phytoplasma* in the roots but not in the rhizosphere of rice samples. In the 18S dataset, we observed that the phylum Nematoda was highly represented in the rice roots, with the genus *Hirschmanniella* (including *H. oryzae*, the rice root nematode) being highly represented in the 18S ASV dataset of the Rovieng samples (higher in roots than in the rhizosphere), while nematodes of the genus *Meloidogyne* (obligatory phytoparasitic nematodes) were present in the Preak Sdei roots and rhizosphere. Oomycota of the genus *Pythium* were also dominant in the 18S ASV of the Preak Sdei roots.

### 3.5 Differential analysis of diseased and healthy samples in root and rhizosphere microbiota

**Figure 5:**
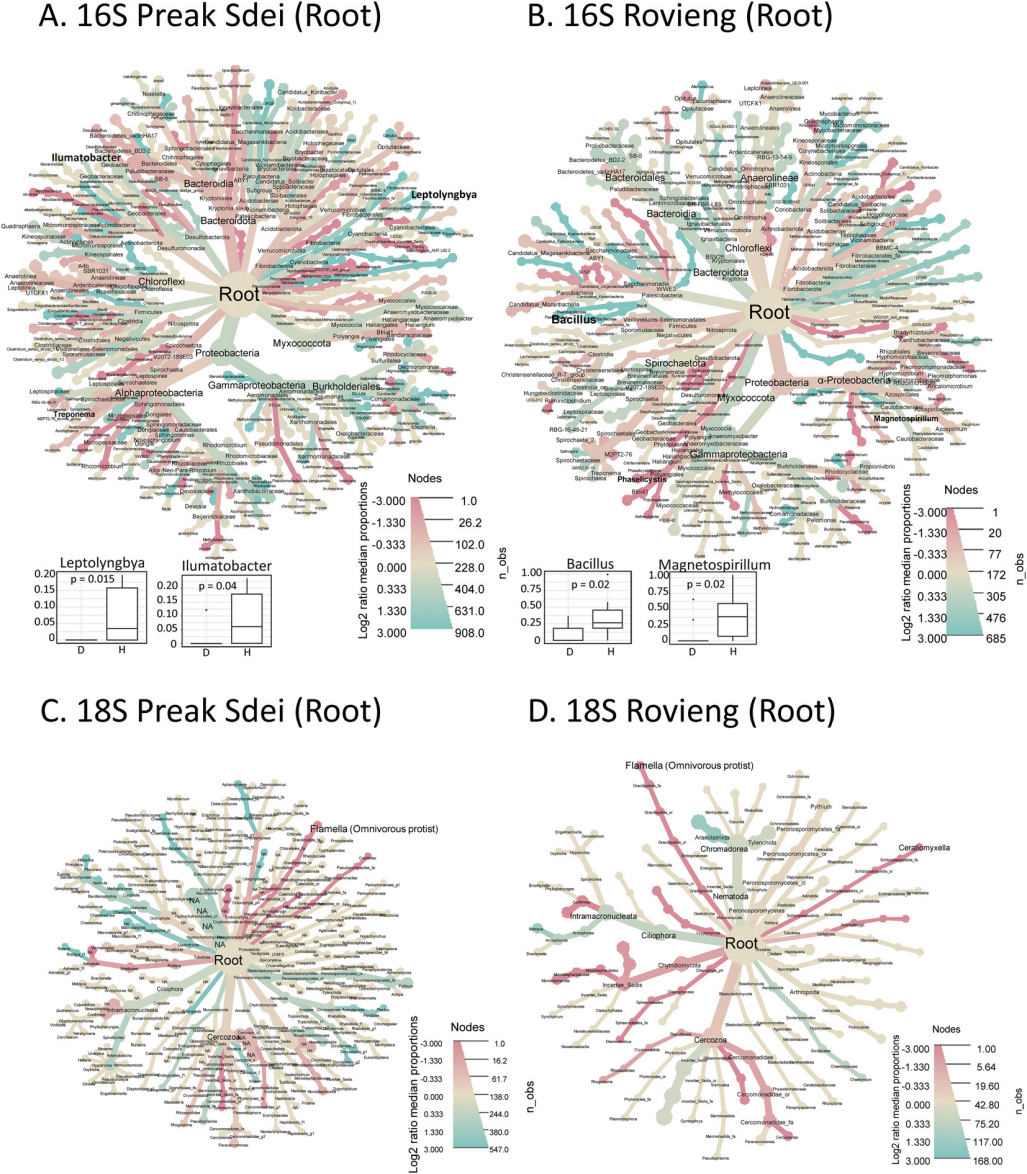
Heat trees showing taxa enrichment between diseased and healthy root samples in 16S ASV **(A, B)** and 18S **(C, D)** amplicon datasets. The heat trees on the left show the analysis on Preak Sdei samples **(A, C)** and on Rovieng on the right **(B, D)**. The colour (blue/red) scale corresponds to the log2 fold enrichment (see colour scale at the bottom right of each heat tree), with blue indicating enrichment in healthy samples and red in diseased samples. The ASV dataset was filtered for read count >50 reads in the sum of all libraries per organ and field to reduce the number of taxonomic ranks shown for easier viewing. Statistical significance between healthy and disease states was assessed using a Wilcoxon test on centre-log ratio normalised datasets. Statistically enriched genera (p<5%) are shown in bold, and when enriched in healthy samples, the box plot is shown at the bottom left of the tree.

**Figure 6:**
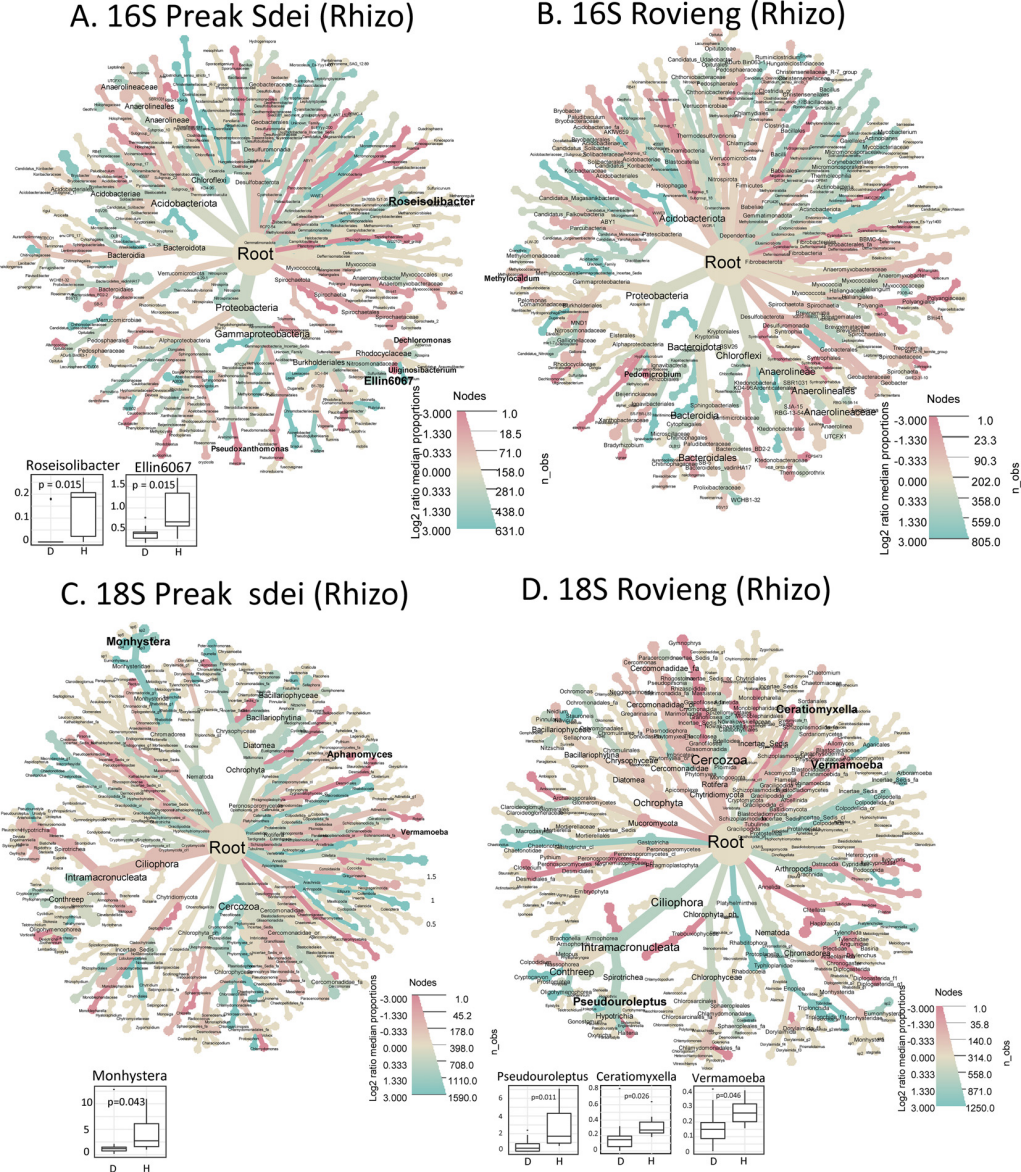
Heat trees showing taxa enrichment between diseased and healthy rhizosphere samples in 16S ASV **(A, B)** and 18S **(C, D)** amplicon datasets. The heat trees on the left show the analysis on Preak Sdei samples **(A, C)** and on Rovieng on the right **(B, D)**. The colour (blue/red) scale corresponds to the log2 fold enrichment, with blue indicating enrichment in healthy samples and red in diseased samples. The ASV dataset was filtered for read count >50 reads in the sum of all libraries per organ and field to reduce the number of taxonomic ranks shown for easier viewing. Statistical significance between healthy and disease states was assessed using a Wilcoxon test on centre-log ratio normalised datasets. Statistically enriched genera (p<5%) are shown in bold, and when enriched in healthy samples, the box plot is shown at the bottom left of the tree.

We performed differential analyses (log2 fold enrichment and Wilcoxon tests on relative abundances) to detect taxa enrichments (genus and species level) in the microbiota of root and rhizosphere samples that correlated with either healthy or diseased leaf status. The heat trees (for 16S and 18S data sets) in the root and rhizosphere are shown in **Figures 5**, **6**, respectively. On these two figures, we have plotted the box plots of microbial genera significantly enriched in healthy samples for each amplicon, field, and compartment. All significantly enriched taxa at genus and species level (either healthy or disease enriched) are available in **Supplementary Figure S5** (16S) and **Supplementary Figure S6** (18S).

In the root samples, several genera appeared to be enriched in healthy samples in log2 fold comparisons (blue colour in the heat trees in **Figures 5A, B**) for the 16S and 18S taxa, but only a few genera could pass the statistical test significance threshold (Wilcoxon test, α=0.05). In Preak Sdei root samples, the bacterial genera *Ilumatobacter* and *Leptolyngbya* and six unnamed genera were enriched in healthy samples (16S ASV dataset, **Figure 5A**), whereas in Rovieng root samples, this was the case for the genera *Bacillus* and *Magnetospirillum* (16S ASV data set, **Figure 5B**). We also detected five genera in Preak Sdei (*Aquabacterium*, *Cloacibacterium*, *Clostridium*, *Shinella*, and one unnamed genus) and five genera in Rovieng (*Phaselicystis* and four unnamed genera) that were significantly enriched in the roots of diseased samples (**Supplementary Figure S5**). For microeukaryotes, there were no significantly enriched genera for the 18S ASV root dataset (healthy or diseased).

In the 16S rhizosphere dataset, the bacterial genera *Roseisolibacter* and *Ellin067* in Preak Sdei and the genus *Iamia* in Rovieng were enriched in healthy samples (**Figures 6A, B**). For the 18S rhizosphere dataset, samples from Preak Sdei showed a statistically significant enrichment of bacterivorous nematodes of the genus *Monhystera* in healthy samples and of the phytopathogenic oomycete genus *Aphanomyces* in diseased samples. Samples from Rovieng showed an enrichment of the protist genera *Pseudouroleptus*, *Coratiomyxella*, and *Vermamoeba* in healthy samples. A dozen unnamed genera were also found either as diseased or healthy enriched (**Supplementary Figure S5**). As we observed high alpha diversity indices in the rhizosphere samples (**Figure 1**), it was unexpected to find so few healthy or diseased signatures in the root and rhizosphere samples. When we plotted the taxonomic binning of each sample for root and rhizosphere samples (**Supplementary Figure S4**), we observed a relatively high variation in taxa distribution between samples of the same condition, which can explain the few detections of healthy- or disease-enriched signatures in statistical analyses. The belowground microbiota appears to be much more diverse and specific to each plant in the field, when analysed at the genus and species level.

### 3.6 Isolation and diversity of *Methylobacterium* and *Methylorubrum* strains

By comparing the microbiota of healthy and diseased samples, we identified a few microbial taxa that were significantly enriched in healthy rice samples compared to diseased ones. In the leaves, a clear signature of bacteria belonging to the genera *Methylobacterium* and *Methylorubrum* was observed. *Methylobacterium* [and *Methylorubrum*, formerly belonging to *Methylobacterium* (Green and Ardley, 2018)] are pink pigmented bacteria that have the specific ability to use C1 sources as carbon sources for growth, including methanol. We therefore exploit this rather rare ability to perform a cultivable approach on leaf samples preserved from our original sampling to isolate bacteria belonging to these two genera. In **Table 2**, we present the different isolated strains belonging to the genera *Methylobacterium* or *Methylorubrum*.

**Figure 7:**
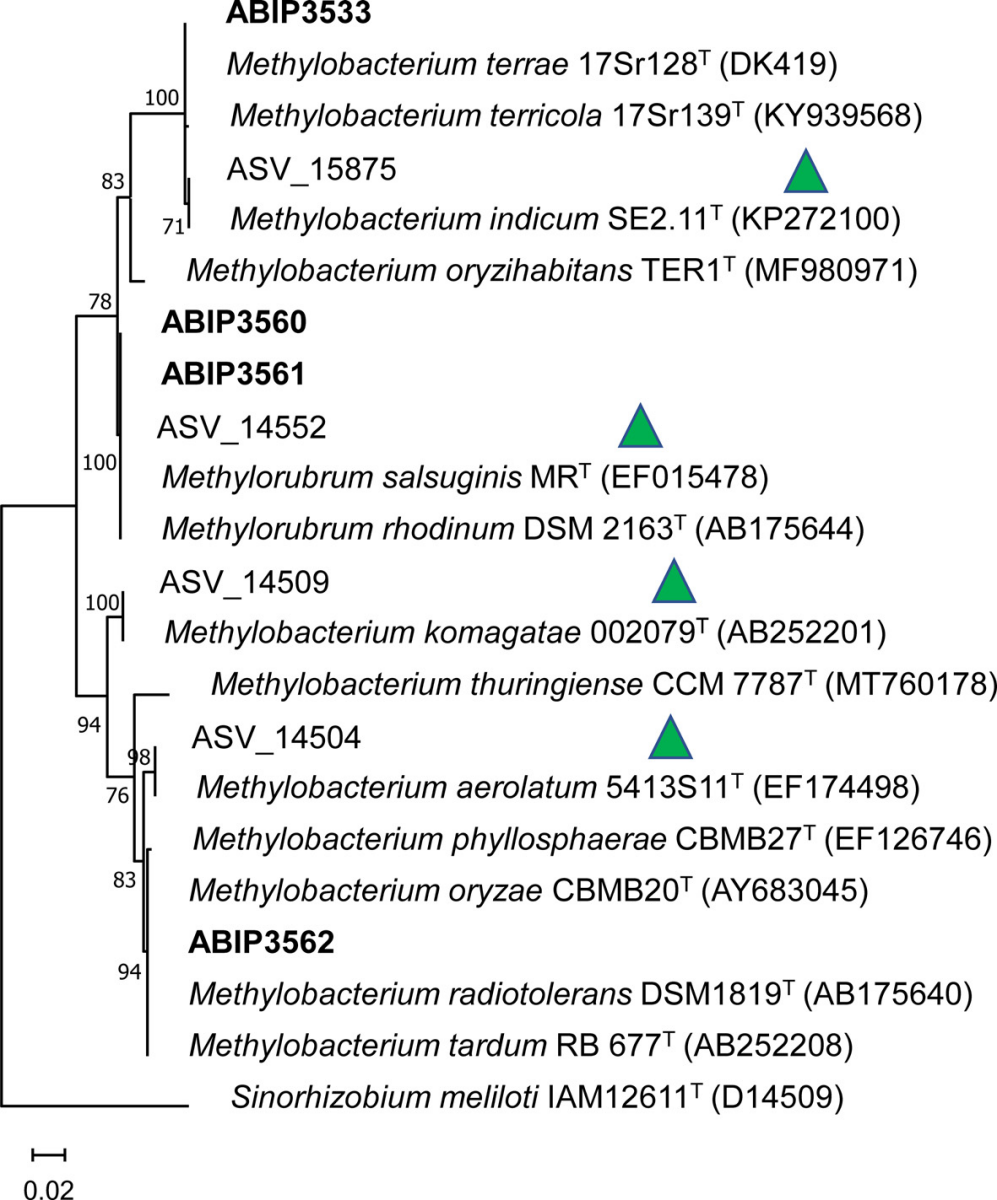
Maximum likelihood tree of the 16S rDNA V3V4 fragment of *Methylobacterium* and *Methylorubrum* strains and ASVs. The phylogeny was obtained by maximum likelihood based on a 400-bp alignment of the V3V4 region of the 16S rDNA. Numbers at tree nodes indicate % of common nodes from a 1,000 bootstrap analysis. Names in bold indicate cultivated strains. Green triangles indicate enriched ASV in healthy leaves. Numbers in parenthesis are NCBI accession numbers (https://www.ncbi.nlm.nih.gov).

We isolated four pink pigmented bacteria from Preak Sdei rice samples (three strains from leaf and one from root). We extracted DNA from these strains and amplified and sequenced a fragment of their 16S rDNA (see *Material and methods*). Blast analyses placed two of them in *Methylobacterium* (ABIP3533 and ABIP3562) and two in *Methylorubrum* (ABIP3560 and 3561). The sequences obtained were compared to the ASV of the microbiome data (only ASV detected as health signature) and closely related species by building a phylogeny based on the partial sequence of the 16S rDNA. A maximum likelihood phylogenetic tree, based on a 400-bp alignment of the V3V4 region of 16S rDNA, is shown in **Figure 7** (method in *Material and methods*), and a distance matrix (% of identity) is shown in **Supplementary Table S5**.

The 16S sequence V3V4 of the isolated strain ABIP3533 shares 99.75% identity with ASV_15875, an ASV found enriched in healthy rice leaves in both Preak Sdei and Rovieng. These sequences group in a clade with *Methylobacterium* (*M*.) *terrae*, *M. terricola*, and *M. indicum*, supported by a 100% bootstrap value (**Figure 7**). The closest species to ABIP3533 is *M. terrae* (97.75% identity), while ASV_15875 shares 100% identity with *M. indicum*. The sequences of strains ABIP 3560 and ABIP3561 share 100% with *Methylorubrum* (Mr) *salsuginis* and ASV_14552 (enriched in healthy samples in Preak Sdei), and they all form a clade with *Mr rhodinum* in the phylogeny. Finally, ABIP3562 groups with a number of species, including *M. oryzae* and *M. phyllosphaerae*, close (99% identity) to ASV1504 (enriched in healthy leaves). Our cultivated strains are thus closely related to the ASV detected by amplicon analysis as enriched in healthy leaves, although they do not always share 100% identity, indicating putative different strains.

### 3.7 Evaluation of biocontrol properties of *Methylobacterium* and *Methylorubrum* strains

**Figure 8:**
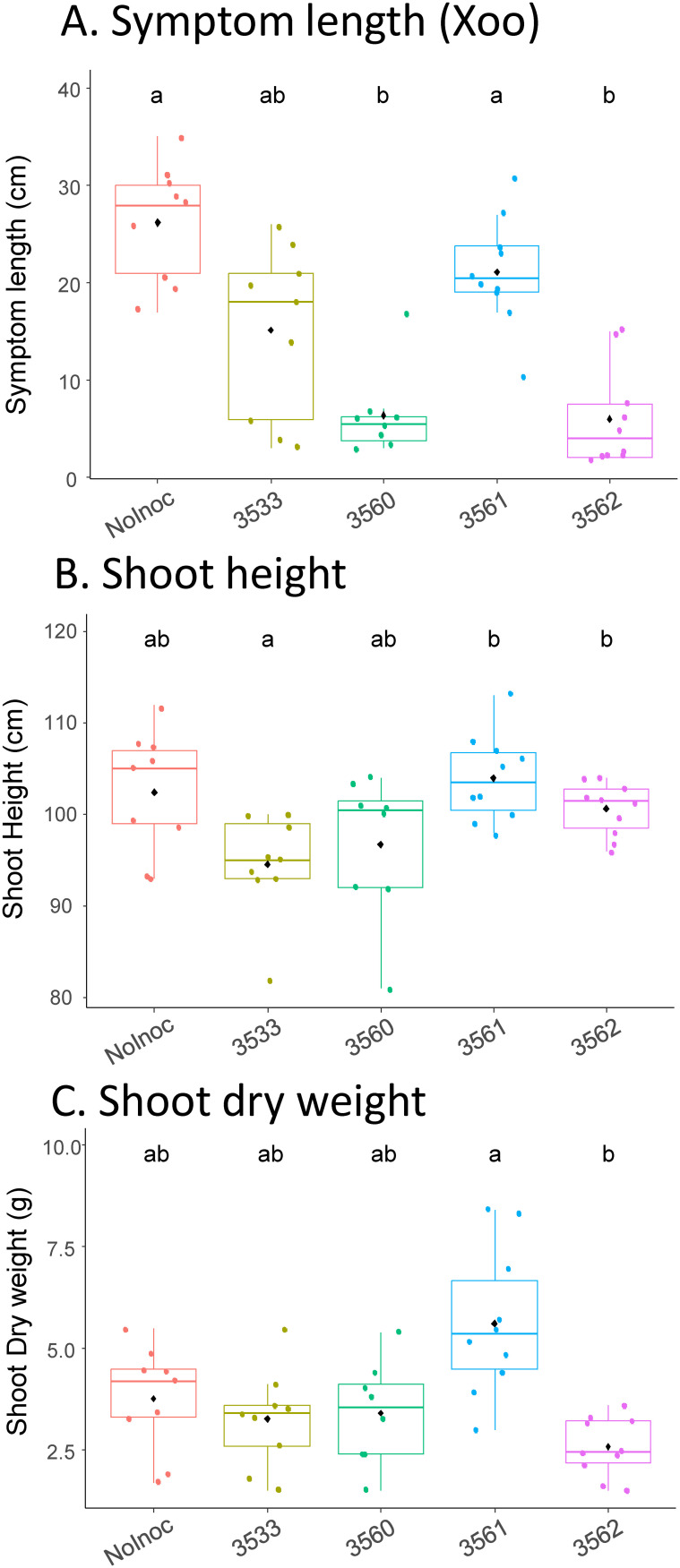
Impact of leaf-spraying inoculation of four methylobacteria strains on symptom size induced by *Xanthomonas oryzae* pv. *oryzae* CIX4551 **(A)**, rice shoot height **(B)**, and rice shoot dry weight **(C)**. Rice leaves (Phka rumduol variety, n=10 per condition) were sprayed at 2 weeks of growth with 10 ml of OD 0.1 bacterial solution of either ABIP3533 (*M. terrae*), ABIP3560 (*Mr rhodinum/salsuginis*), ABIP3561 (*Mr rhodinum/salsuginis*), ABIP3562 (*M. aerolatum/oryzae*), or with water (control, “NoInoc” in figure). Plants were leaf-clipped at 4 weeks with *Xoo* CIX4551 (virulent strain isolated from rice in Cambodia), and symptoms measured at 6 weeks. Letters above box plots are statistical groups from a pairwise Wilcoxon test between conditions (α=5%, Bonferroni correction).

Since we have isolated *Methylobacterium* and *Methylorubrum* strains that are taxonomically close to significantly enriched ASV in healthy rice leaves, we tested the hypothesis that these strains might have a bioprotective capacity against the infection by a rice phytopathogen. We used the *Xanthomonas oryzae* pv. *oryzae* (*Xoo*) pathosystem, with a *Xoo* strain (CIX4551) isolated in Cambodia from bacterial leaf blight symptoms on Phka rumduol rice leaves and shown to be virulent on this variety in previous laboratory tests. This pathosystem has the advantage of allowing a quantitative assessment of symptoms by measuring symptom size from the clipping infection site. We sprayed Phka rumduol leaves (10 plants per strain) with two strains of *Methylobacterium* (ABIP3533 and ABIP3562) and two strains of *Methylorubrum* (ABIP3560 and ABIP3561), at 2 weeks of growth (See *Material and methods*). The control condition was Pkha rumduol leaves sprayed with sterile distilled water. The *Xoo* strain was leaf clipped at 4 weeks of growth, and symptom size, shoot height, and shoot dry weight were monitored at 6 weeks of growth. Results are shown as box plots in **Figure 8**, and mean differences were assessed with a Kruskal–Wallis test followed by pairwise Wilcoxon tests across conditions (α=5%). Leaf inoculation with ABIP3560 and ABIP3562 significantly reduced the symptom size of *Xoo* infection compared to the control condition (**Figure 8A**), in contrast to ABIP3533 and ABIP 3561. The reduction in symptom size was 75% and 77% for ABIP3560 and ABIP3562, respectively, compared to the control condition. Despite 100% identity in their 16S partial sequence, strains ABIP3560 and ABIP3561 showed different bioprotective phenotypes. Differences were observed for shoot height (**Figure 8B**), with ABIP3533 inoculation giving significantly lower plants than with ABIP3561 and ABIP3562, while for shoot dry weight (**Figure 8C**), only ABIP3561 inoculation differed from ABIP3562. Thus, spray inoculation of rice leaves with our isolated strains had different effects on shoot development and pathogen infection.

## 4 Discussion

### 4.1 Comparing the microbiota of diseased and healthy rice reveals a clear health signature in the leaves, but not in the roots or rhizosphere

Plants are considered to be holobionts (Vandenkoornhuyse et al., 2015) because they live with a variety of microbes on and in their tissues. Numerous studies have described the rice microbiome belowground (Edwards et al., 2015; Ding et al., 2019; Kim and Lee, 2020; Guo et al., 2021) and in the phyllosphere (Roman-Reyna et al., 2019), and direct correlations have been found between the presence of specific taxa and disease resistance in rice (Liu et al., 2023). However, few studies have tested the hypothesis that healthy plants in fields under high pathogen pressure (surrounded by diseased plants) may be protected by specific microbial taxa. Describing the specific microbiota of healthy plants in this context could shed light on the composition of the bioprotective microbiota and identify specific microbial taxa involved in this capacity. This hypothesis is supported by the fact that plants can recruit specific microbial taxa for their protection (Liu et al., 2021) and that this recruitment can be modulated by airborne signals (volatiles) from diseased plants (Li et al., 2021).

In this study, we analysed the composition of the rice microbiota between diseased and healthy plants in two rice fields with a high leaf disease symptom rate, looking for microbial taxa as signatures of health. After describing which phytopathogens were present in our diseased samples, we compared the microbiota composition of leaves, roots, and rhizosphere between healthy and diseased rice plants. We chose the 16S V3V4 amplicon to profile bacteria and the 18S V4 to profile all microeukaryotes. We preferred the 18S to the more fungus-specific ITS amplicon because we wanted to profile all microeukaryotes, including nematodes and protists. If the 16S V3V4 performed well in all plant compartments for bacterial genus and species profiling at the ASV level, the 18S V4 produced very few microbial sequences in the leaves but performed well in the roots and rhizosphere, allowing the detection of fungi, protists, and nematodes (including rice phytoparasitic nematodes belonging to the genera *Meloidogyne* and *Hirschmanniella*). However, the low diversity of the 18S amplicon did not allow the detection of clear signatures in the microeukaryotes at the family or genus level.

We found a clear health taxa signature in the leaf microbiota of ASVs of the genus *Methylobacterium*, which was conserved in the two fields, and of *Methylorubrum* ASVs in Preak Sdei. It is noteworthy that the two fields studied were unrelated both geographically (Preak Sdei and Rovieng are 330 km apart), in terms of the rice variety used (first cycle premium jasmine rice versus second cycle local indica variety), soil composition, and pests and pathogens diversity (**Table 3**). We also found an enrichment of bacterial species in leaf diseased samples, including the genera *Pantoea*, *Sphingomonas*, and *Pseudomonas*, which host phytopathogenic species of rice, highlighting the presence of multiple bacterial pathogens in the fields. *Xanthomonas oryzae* was found in both healthy and diseased samples, as confirmed by diagnostic PCR and was therefore not enriched in diseased samples. There were also many non-pathogenic bacterial genera that were enriched in leaf diseased samples and could constitute the rice pathobiota (taxa associated with reduced health status). Some of these were common to both fields, such as *Aureimonas* sp., *Hymenobacter* sp., *Mucilaginibacter* sp., or *Spirosoma* sp. (**Supplementary Figure S3**). The presence of several fungal pathogens on leaves was also detected by diagnostic PCR, but we could only monitor the expected fungal pathogens (narrow brown spot, brown spot, and blast) because the 18S V4 amplicon did not work for leaves. In the case of roots and rhizosphere, only a few bacterial taxa could be identified as being enriched in healthy samples. In Preak Sdei, healthy roots were enriched in cyanobacteria of the genus *Leptolyngbya* and actinobacteria of the genus *Ilumatobacter*. Although a strain of *Leptolyngbya* has been described to promote plant growth in sunflower (Mao et al., 2024), the bibliography on the association of *Ilumatobacter* with plants is scarce, apart from its description as an abundant genus in the rhizosphere of halophytes (Yamamoto et al., 2020). In the Preak Sdei rhizosphere, healthy samples were enriched in *Roseisolibacter* and *Ellin6067*. The genus *Roseisolibacter* contains only one described species, *R. agri*, isolated from agricultural floodplain soils, and there is no information on its potential PGPR capacities. For the genus *Ellin6067*, it has been reported as an abundant genus in various rice microbiota studies (Dong et al., 2023; Jiang et al., 2024), but no article describes its potential PGPR capacities, as there was no cultured strain until recently (Verhoeven et al., 2023). In Rovieng healthy root samples, the genera *Bacillus* and *Magnetospirillum* were enriched. *Bacillus* is well known to contain many PGPR species for rice, from modification of rice root architecture development (Ambreetha et al., 2018), abiotic stress alleviation (Siddika et al., 2024) to biocontrol of rice diseases (Pandey et al., 2024), to cite a few. Unfortunately, the taxonomic resolution of the 16S V3V4 amplicon sequence did not allow us to determine which species were specifically enriched, as the 16S V3V4 is not informative for this particular genus (Xu et al., 2023). For *Magnetospirillum*, this genus has been described as abundant in the rice microbiota and able to degrade toluene originating from root exudates (Lu et al., 2006), but no strains from this genus have been described as involved in biocontrol or stimulation of plant defence. For the 18S V4 amplicons, we found an enrichment of the nematode genus *Monhystera* in Preak Sdei and of the protist genera *Pseudouroleptus* (ciliate, phylum Ciliophora), *Ceratiomyxella*, and *Vermamoeba* (both from phylum Amoebozoa) in Rovieng, in the rhizosphere of healthy samples. Nematodes of the genus *Monhystera* feed on bacteria and may play a role in modifying the rhizosphere microbiota. For example, increased rhizosphere biodiversity in conservation agriculture in rice was found to modify the soil food web, enriching functional diversity of nematodes (including bacterivores) and reducing phytoparasitic nematodes (Masson et al., 2022). Plant-associated protists have also been described to play a beneficial role in plant (including rice) growth and biocontrol of diseases (Nguyen et al., 2023), although the enriched genera found in this study had not been identified in previous studies, highlighting a putative role for these protist taxa in rice health. For taxa enriched in the roots or rhizosphere of diseased samples, no phytopathogen could be detected (even the phytoparasitic nematodes that present on all plants) except the oomycete *Aphanomyces* in Preak Sdei, and none were common to both Rovieng and Preak Sdei, reflecting a pathobiota specific to each site. We monitored the presence of pathogens in our amplicon sequences generated from leaves, roots, and rhizosphere samples from two fields with contrasting practices, soils, and varieties. As we only had one field in each situation, we cannot draw correlations between practices, varieties, and disease presence. However, we did observe different patterns of pathogen presence, revealing contrasting pathogen pressures; for example, the phytoparasitic nematode *Hirschmanniella oryzae* is highly abundant in Rovieng, whereas *Meloidogyne graminicola* is common in Preak Sdei. Developing our approach across a number of fields using the same practices and varieties should help to reveal their impact on disease incidence and rice microbiota.

### 4.2 *Methylobacterium* and *Methylorubrum* as signatures of rice health in the phyllosphere

In our study, we found that the genera *Methylobacterium* and *Methylorubrum* were enriched in the leaves of healthy rice. These two genera include species that belong to a group called pink-pigmented facultative methylotroph (PPFM), which are commonly found in soil, dust, water, and the phyllosphere (Omer et al., 2004). Their pigmentation and ability to use methanol as their sole source of carbon and energy make PPFM particularly well adapted to colonise and survive on leaves under these harsh conditions (UV light, low substrate). Indeed, growing plant cells emit significant amounts of methanol, due to their wall-associated pectin metabolism (methanol is a derivative of pectin degradation). These capabilities have led to the hypothesis that plant-associated methylobacteria have co-evolved with plants as phytosymbionts (Kutschera, 2007). Our result underlines the fact that either healthy leaves are able to host more PPFM compared to diseased leaves and/or that these PPFM can protect the plant against phytopathogen spread and disease. It has also been reported that the emission of methanol by a wounded plant increases the resistance of the unwounded neighbouring plants to bacterial pathogens by activating methanol-inducible genes (Komarova et al., 2014; Dorokhov et al., 2018). As we sampled rice in fields with a high disease rate (looking for healthy rice surrounded by symptomatic rice), we can also hypothesise that high rates of methanol were emitted by diseased plants, making methylobacteria more abundant on healthy leaves, as their intact surfaces would provide more space for colonisation. Thus, the higher abundance of methylobacteria in healthy leaves could simply be a consequence of the diseased environment in the field. Another simple explanation would be the undegraded surface of healthy leaves compared to diseased ones, but if this were the case, we should have detected an enrichment of other common leaf inhabitants such as *Pantoea* or *Pseudomonas*, which are part of the core microbiome of the rice phyllosphere (Roman-Reyna et al., 2019; Yin et al., 2023).

Our ASV analysis allowed us to identify three *Methylobacterium* species (*M. indicum* in both fields, *M. komagatae*, and *M. aerolatum* in Preak Sdei) and one *Methylorubrum* species (*Mr. rhodinum* in Preak Sdei) as health signature taxa. It has been reported that the site and plant species are important drivers of methylobacteria leaf communities, so it was expected that differences would be found in both fields (Knief et al., 2010). Nevertheless, the species *M. indicum* was detected as a health signature in both plots. *M. indicum* was originally isolated from surface-sterilised rice seeds (Chaudhry et al., 2016), but recent work has described some strains of this species as rice phytopathogens causing leaf bleaching in Vietnam, although the type strain of the species is not pathogenic (Lai et al., 2021). Therefore, this species does not appear to be a good candidate for biocontrol of rice phytopathogens. Unfortunately, we did not isolate a strain of *M. indicum* in our cultivable approach to test its phytopathogenic or biocontrol potential. However, a closely related strain (ABIP3533) belonging to the species *M. terrae/M. terricola* was obtained (**Figure 7**), which was not pathogenic to rice in our tests. The species *M. komagatae* and *M. aerolatum* have also been reported in the rice phyllosphere microbiome (Tani et al., 2015; Lai et al., 2020). Finally, the *Methylorubrum rhodinum* species has not been reported in the rice microbiome, except a mention in Lai et al. (2020) as *M. rhodium*, which is probably a misspelling. We did not identify the *M. oryzae* species as a health taxa signature, despite the fact that this species is well documented as a rice PGPR (Kwak et al., 2014; Walitang et al., 2023), is abundant in our 16S ASV dataset (ASV_14496, **Supplementary Table S3**), and we were able to isolate a strain of this species.

### 4.3 *Methylobacterium* and *Methylorubrum* strains as promising bioinoculants for sustainable rice production

Many articles have reported the plant-growth-promoting and biocontrol capacities of *Methylobacterium* species and their potential as bioinoculants for sustainable agriculture (reviewed in (Dourado et al., 2015; Zhang et al., 2021). In order to gain further insight into a putative causality between methylobacteria enrichment and the rice health status, we evaluated the hypothesis that these bacteria could protect rice against a phytopathogen attack, by isolating four strains (two *Methylobacterium* and two *Methylorubrum*) and testing their bioprotection by leaf spraying on rice (on Cambodian Phka rumduol variety), using the *Xoo* pathosystem for the quantitative assessment of bacterial leaf blight symptoms. We found that a strain of *Methylorubrum* (ABIP3560, close to *Mr. rhodinum* and *Mr. salsuginis*) and a strain of *Methylobacterium* (ABIP3562, close to *M. aerolatum* and *M. oryzae*) significantly reduced the symptoms of *Xoo* leaf infection (−75% of symptom size compared to the control), whereas ABIP3533 (*M. terrae*, which is not a health-signature taxon, **Figure 3**) and ABIP3561 (a strain with 100% identity in 16S with ABIP3560) did not. The bioprotective effect of methylobacteria inoculation could have multiple origins as described in the literature, ranging from activation of plant defences through upregulation of pathogenesis-related gene expression via various plant hormone pathways (Yang et al., 2024), to modification of the plant microbiota (Ardanov et al., 2012), to direct antagonism against phytopathogens (Vadivukkarasi and Bhai, 2020). We are currently sequencing the complete genomes of these strains to get further insight into their ability to biocontrol rice phytopathogens and additional PGPR traits. We will also test the bioprotective effect of these strains against other phytopathogens, as plants hosting methylobacteria as health signatures were under multiple phytopathogen pressure in the sampled fields (phytoplasma, narrow brown spot, and bacterial leaf blight). Field inoculation tests should be also conducted to evaluate the bioprotective capacities at field level and in different agronomic practices. Field tests have already been described by seedling inoculation of different *Methylobacterium* strains, showing for some of them a positive impact on growth and yield (Sanjenbam et al., 2022). Given the phenotypes observed in our study, evaluating the bioprotective impact of methylobacteria should be conducted to evaluate their potential as bioprotective inoculants against various rice phytopathogens.

### 4.4 Conclusion

The analysis of the microbiota between leaf diseased and healthy rice has identified the genera *Methylobacterium* and *Methylorubrum* as health taxa signatures, including different species. A culturable approach using methanol as sole-carbon source allowed to isolate several strains that proved to reduce disease symptoms of bacterial leaf blight when sprayed on rice leaves. We thus validated the hypothesis that healthy plants in fields under high disease occurrence can host specific microbiota with biocontrol capacities. This strategy could help identify new microbes with biocontrol potential for sustainable rice production. Further description of the strains at the genome level, bioprotective capacity against a range of phytopathogens, plant colonisation, and impact on plant health will be undertaken to validate their potential as sustainable alternatives for rice disease management in Cambodia.

## Data Availability

The amplicon sequencing data (fastq) for this study are accessible in the ENA (European Nucleotide Archive, https://www.ebi.ac.uk/ena) database under the Bioproject PRJEB70289 (ERP155223).
