# Supplementary material for: Comparison of plant microbiota in diseased and healthy rice reveals methylobacteria as health signatures with biocontrol capabilities
Source: Front Plant Sci. 2024 Oct 29;15:1468192. doi: 10.3389/fpls.2024.1468192 (PMC11554501; doi:10.3389/fpls.2024.1468192)

Supplementary Figure S1: Neighbor joining of 18S V4 fragment of nematoda reference sequences and 18S amlicons nematoda ASV from this study.

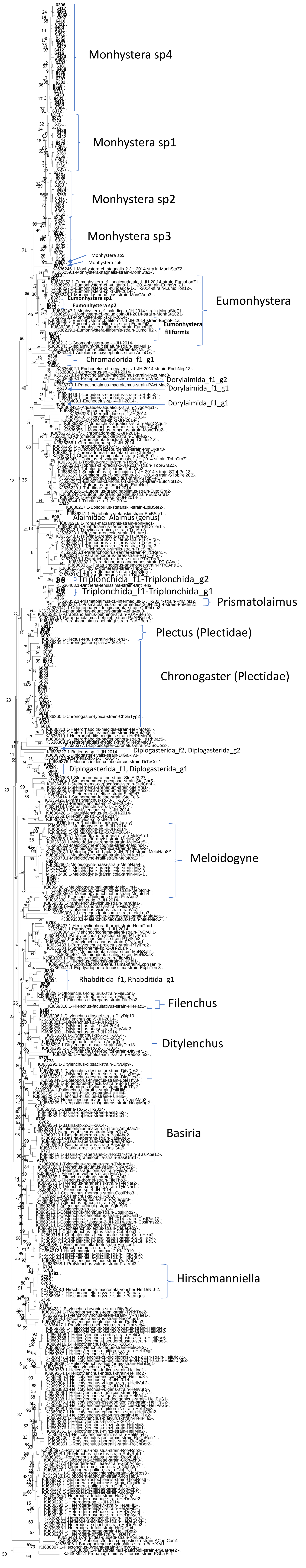

Supplement: Supplementary file 1 [file DataSheet1.pdf]
