# Supplementary material for: Comparison of plant microbiota in diseased and healthy rice reveals methylobacteria as health signatures with biocontrol capabilities
Source: Front Plant Sci. 2024 Oct 29;15:1468192. doi: 10.3389/fpls.2024.1468192 (PMC11554501; doi:10.3389/fpls.2024.1468192)

16S NMDS

PREAK SDEI LEAVES

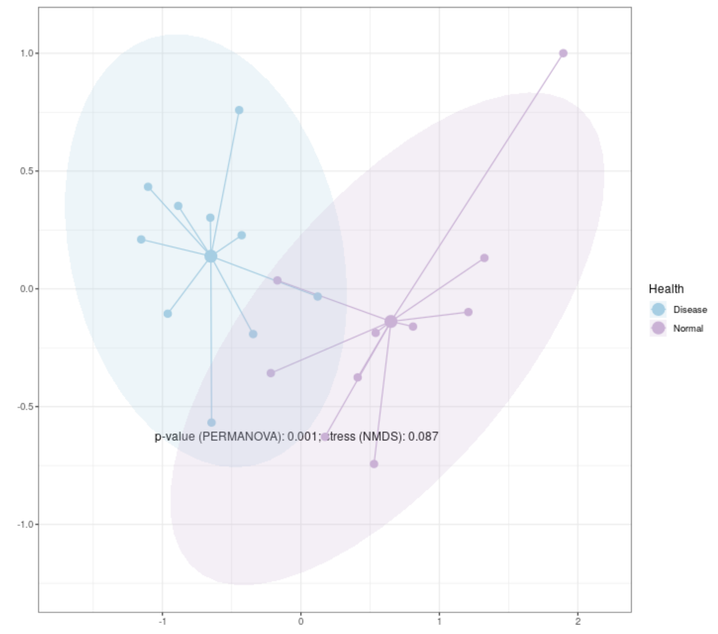

ROVIENG LEAVES

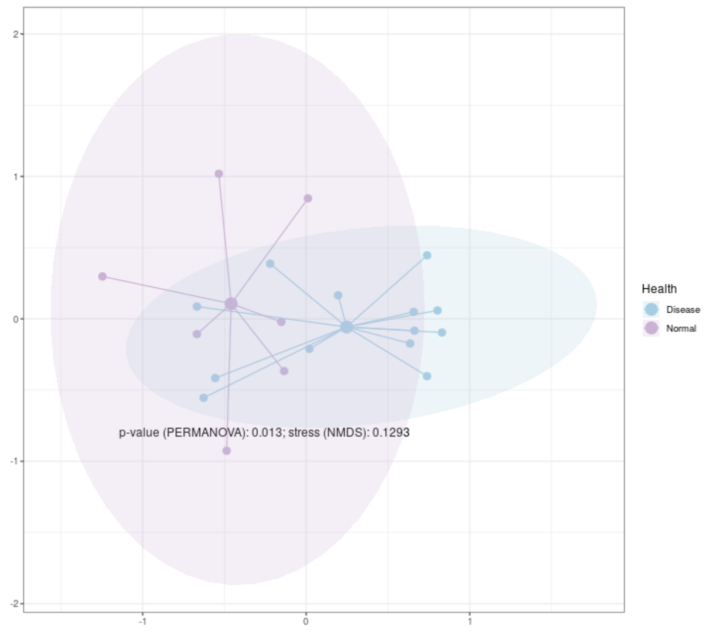

PREAK SDEI RHIZOSPHERE

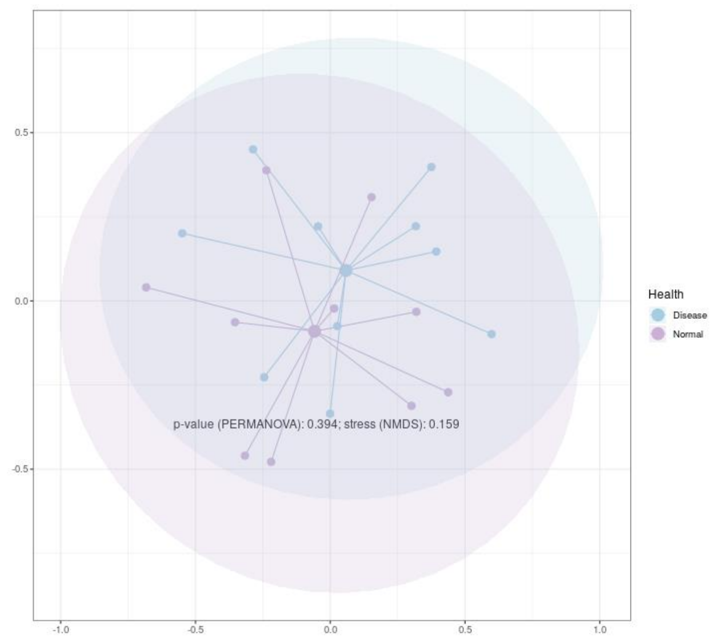

14219 GGAATATTG[illegible]

ROVIENG RHIZOSPHERE

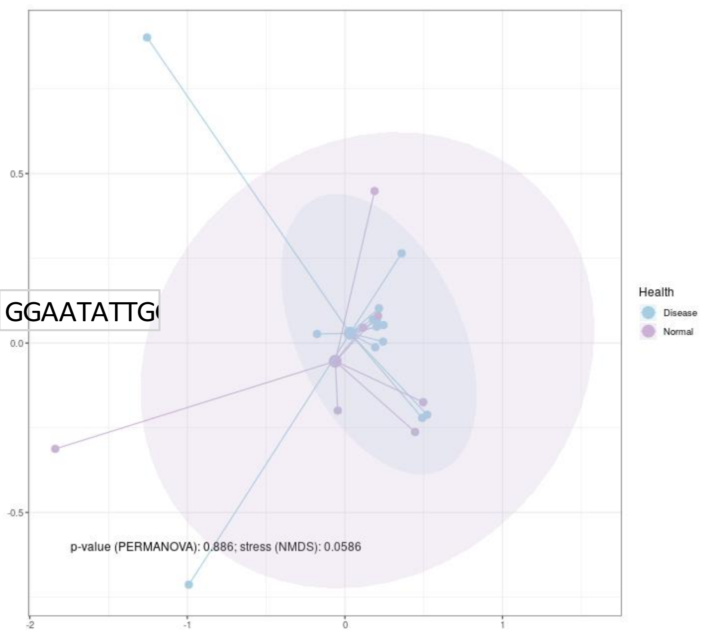

PREAK SDEI ROOT

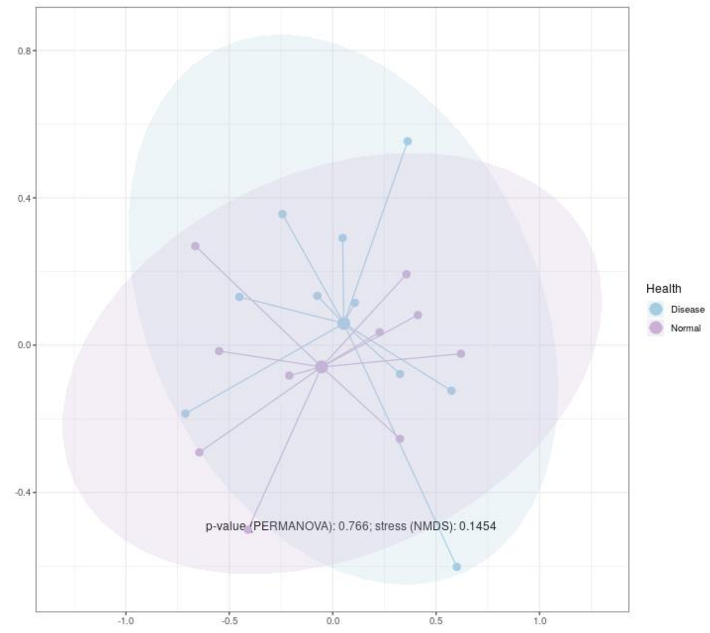

ROVIENG ROOT

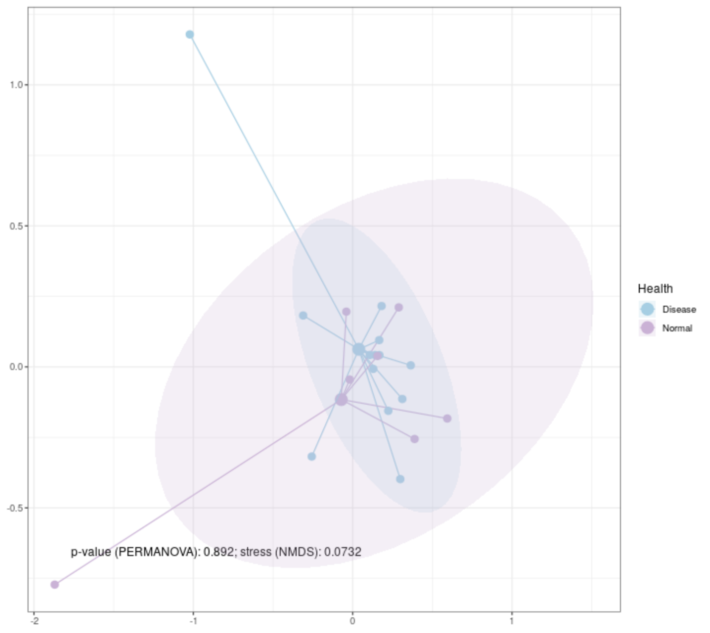

18S NMDS

PREAK SDEI RHIZOSPHERE

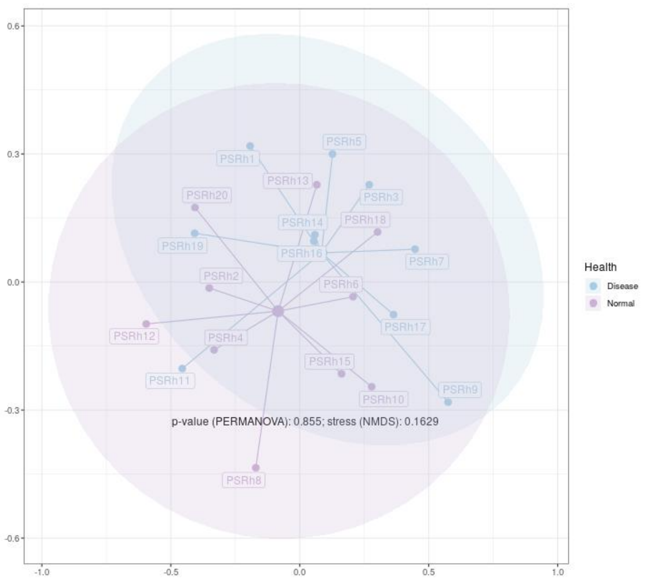

ROVIENG RHIZOSPHERE

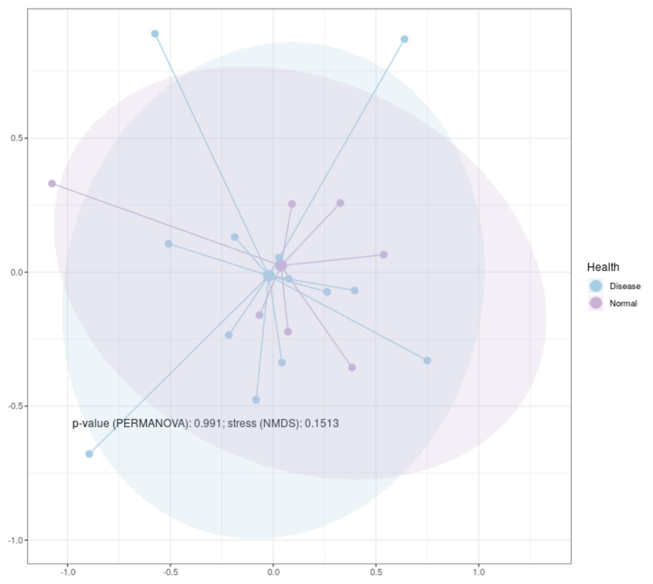

PREAK SDEI ROOT

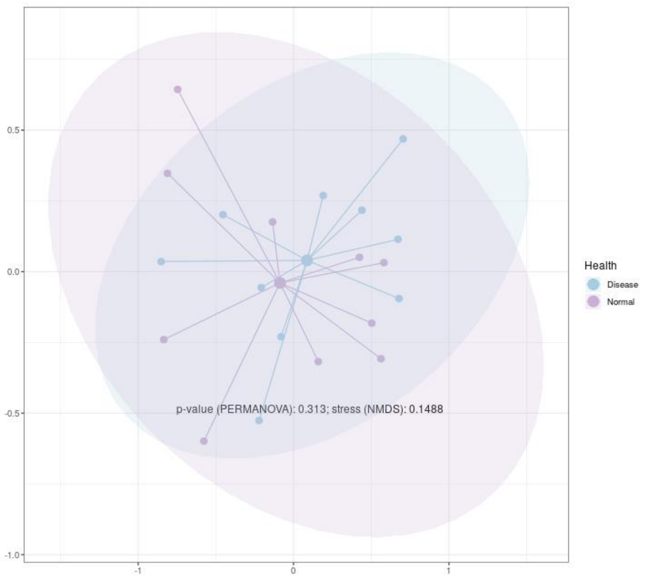

ROVIENG ROOT

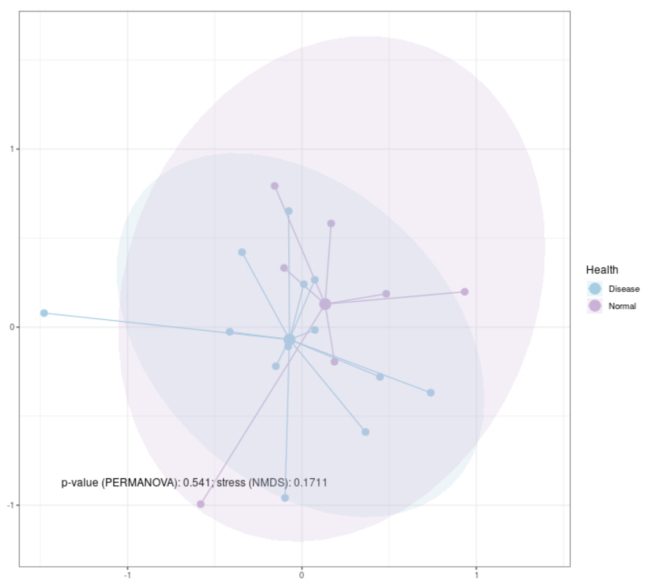

Supplement: Supplementary file 2 [file DataSheet2.pdf]
