# Supplementary material for: Comparison of plant microbiota in diseased and healthy rice reveals methylobacteria as health signatures with biocontrol capabilities
Source: Front Plant Sci. 2024 Oct 29;15:1468192. doi: 10.3389/fpls.2024.1468192 (PMC11554501; doi:10.3389/fpls.2024.1468192)

Supplementary Figure S3A:
Preak Sdei enriched genera in healthy and diseased samples

Relative abundance were normalised by center-log ratio method. To detect enriched taxa, a wilcoxon test (alpha=0.05; Bonferroni correction) was conducted between disease and healthy (normal) samples.

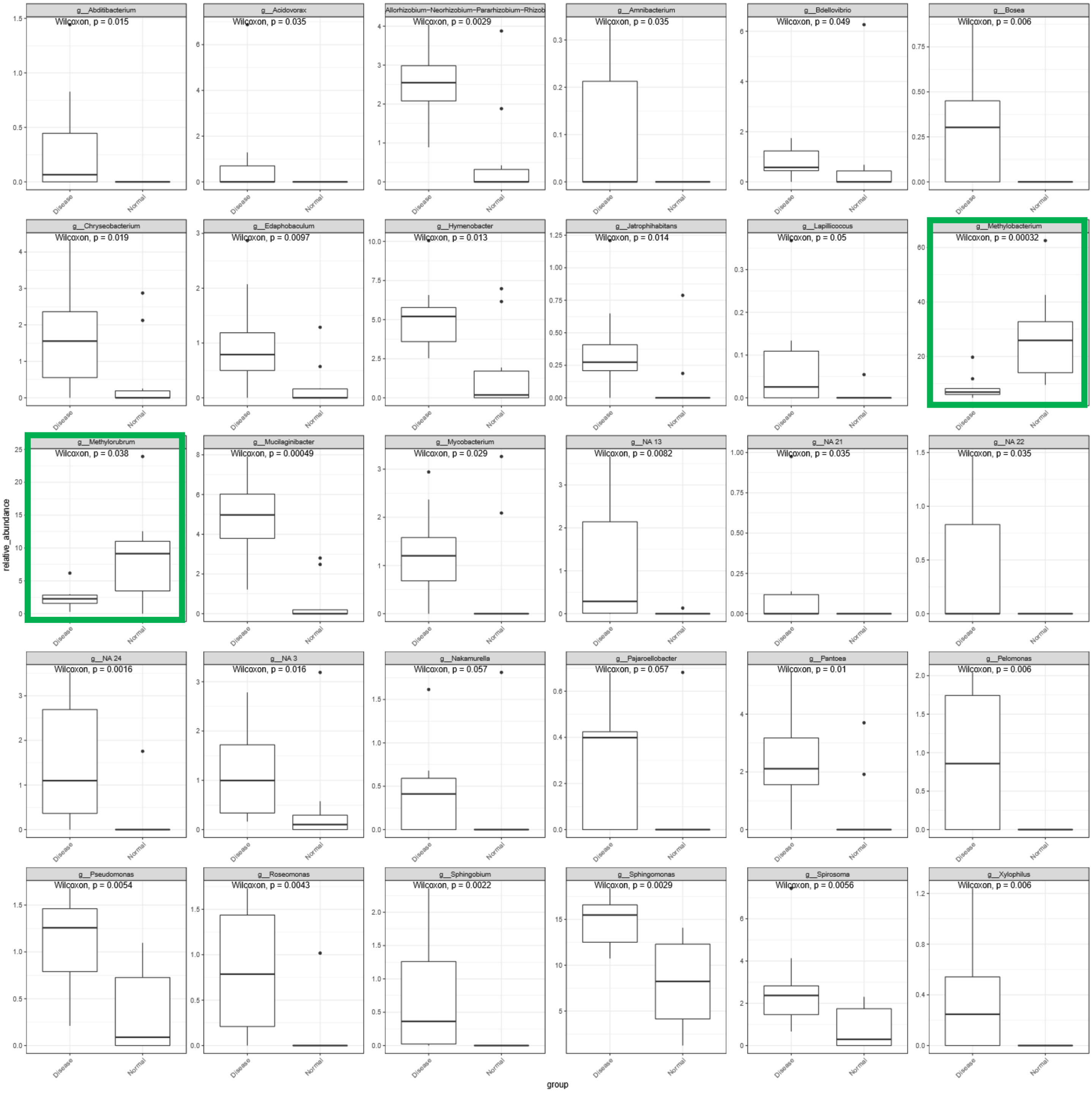

Supplementary Figure S3A:
Preak Sdei enriched <u>species</u> in healthy and diseased samples

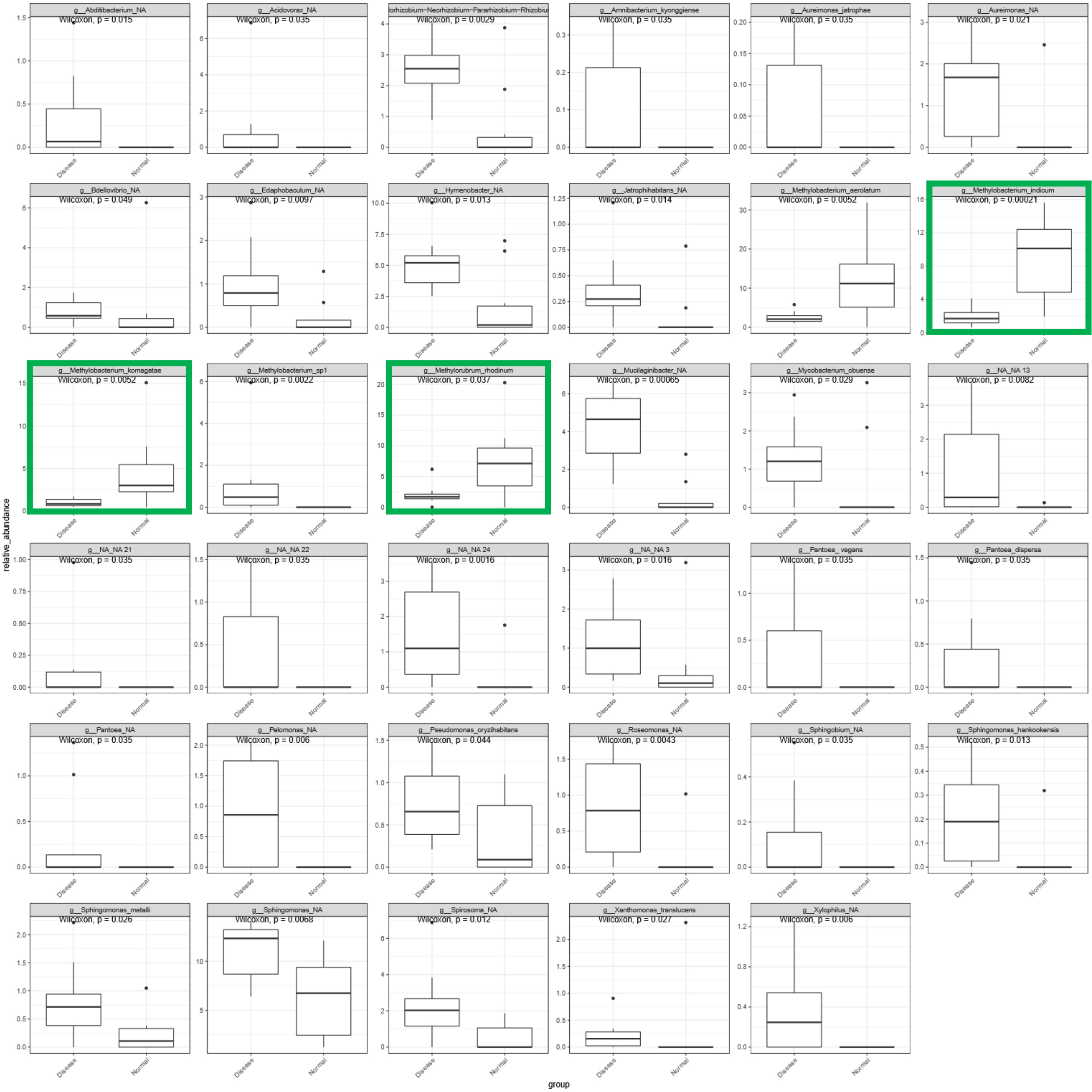

Supplementary Figure S3B:
Rovieng enriched genera in healthy and diseased samples

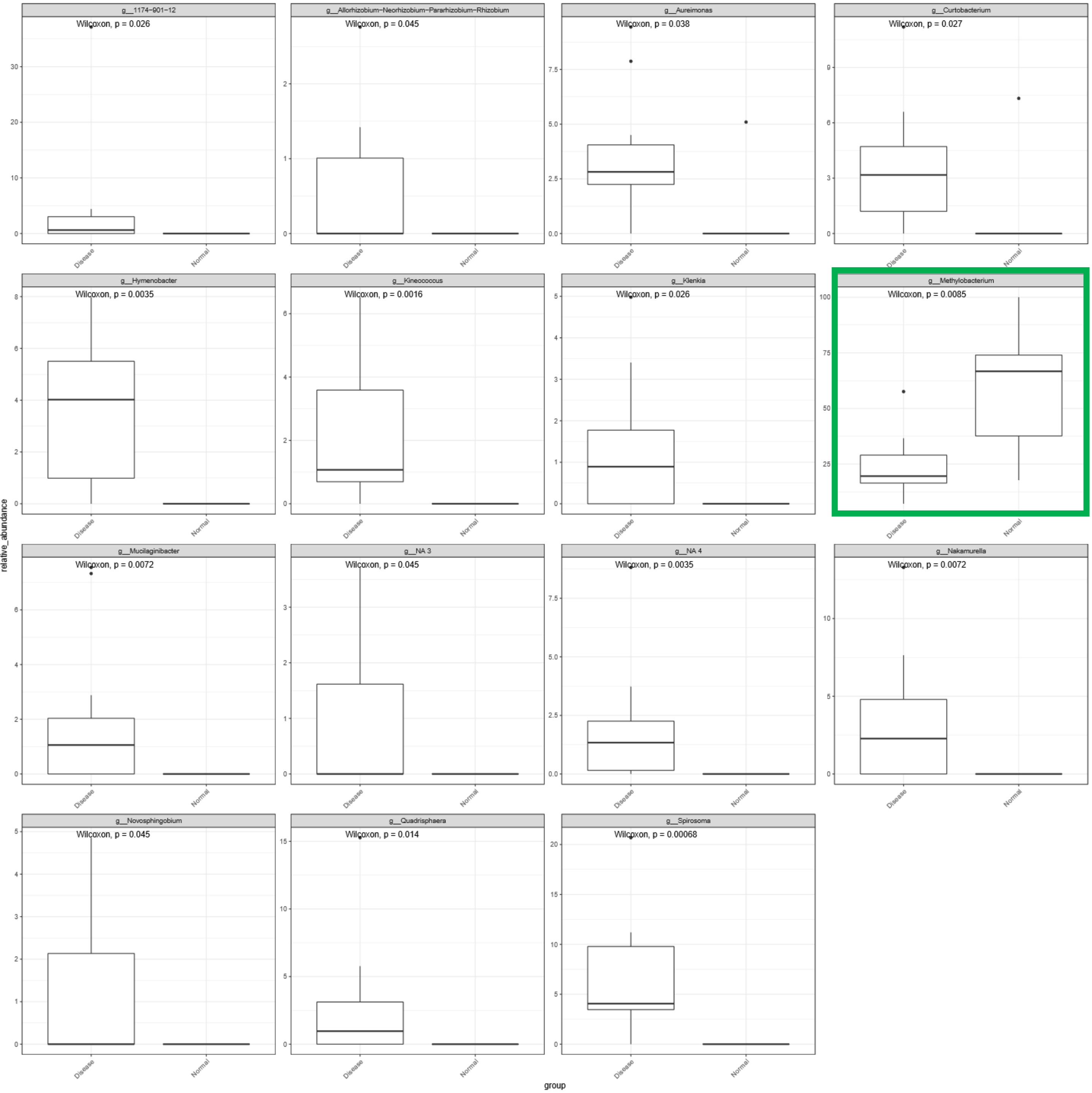

Supplementary Figure S3B:
Rovieng enriched species in healthy and diseased samples

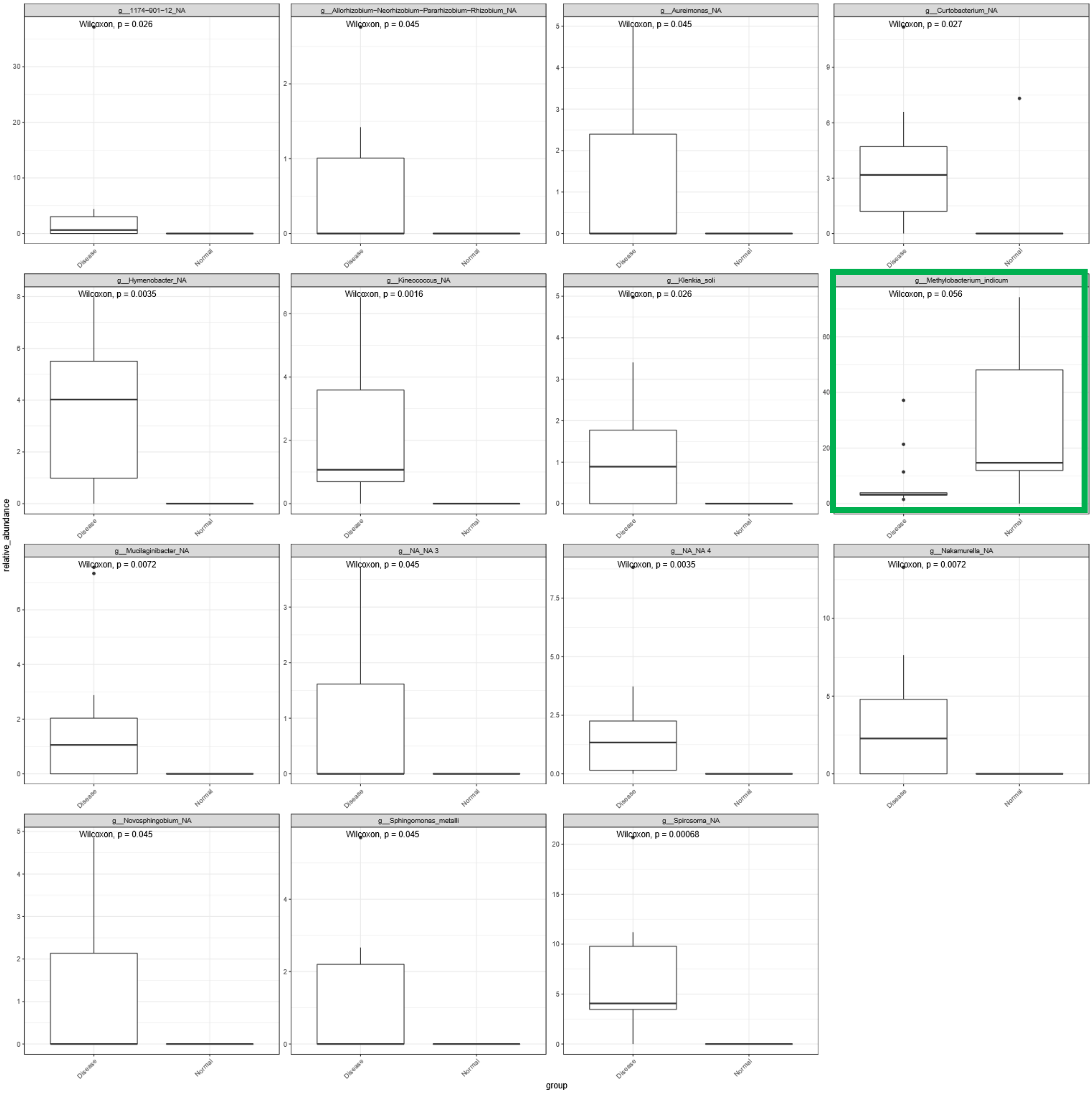

Supplement: Supplementary file 3 [file DataSheet3.pdf]
