# Supplementary material for: Comparison of plant microbiota in diseased and healthy rice reveals methylobacteria as health signatures with biocontrol capabilities
Source: Front Plant Sci. 2024 Oct 29;15:1468192. doi: 10.3389/fpls.2024.1468192 (PMC11554501; doi:10.3389/fpls.2024.1468192)
Supplement: Supplementary file 4 [file DataSheet4.pdf]

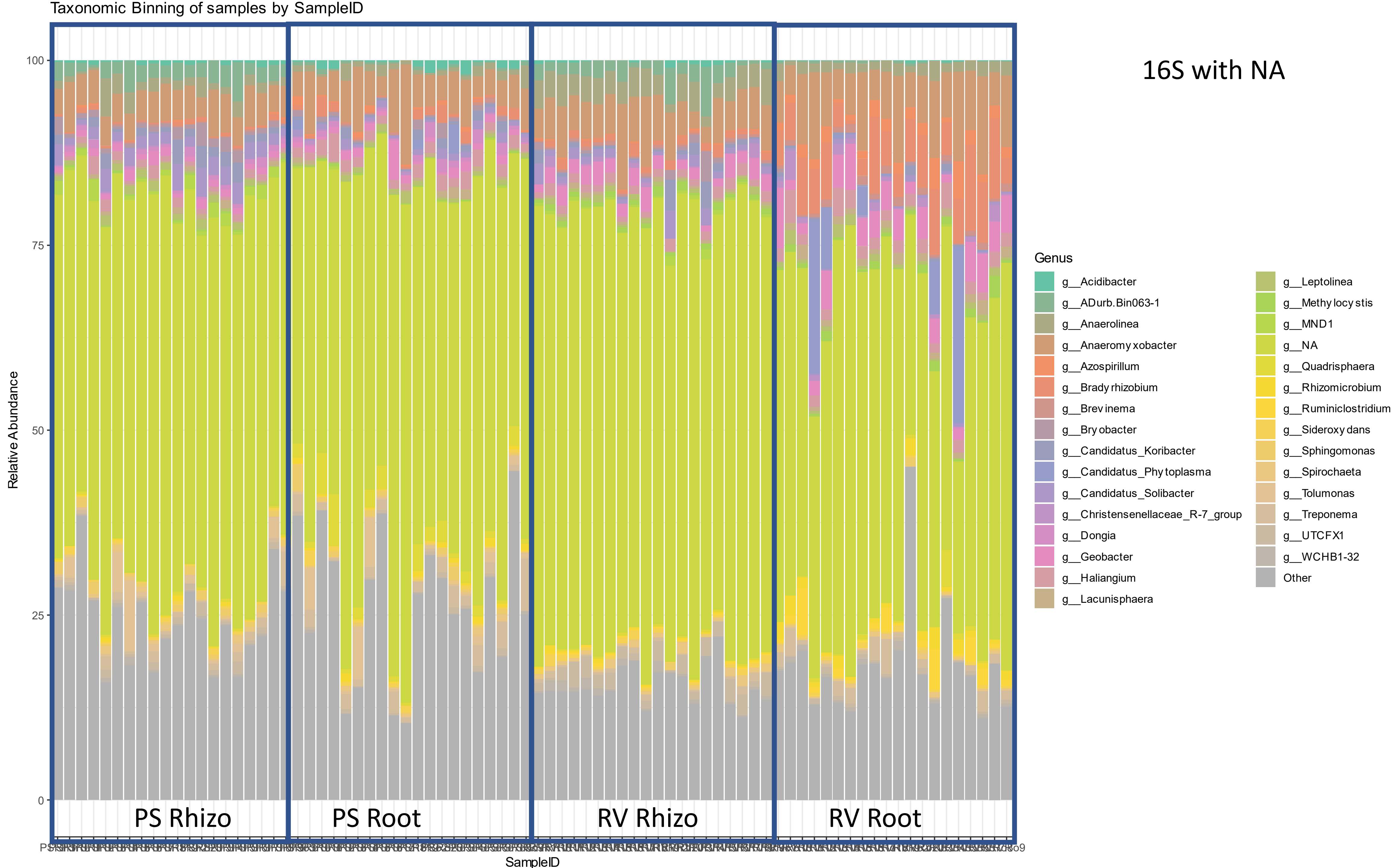

Taxonomic Binning of samples by SampleID
16S with NA
Relative Abundance
100
75
50
25
0
SampleID
PS Rhizo
PS Root
RV Rhizo
RV Root
Genus
g__Acidibacter
g__ADurb.Bin063-1
g__Anaerolinea
g__Anaeromy xobacter
g__Azospirillum
g__Brady rhizobium
g__Brev inema
g__Bry obacter
g__Candidatus_Koribacter
g__Candidatus_Phy toplasma
g__Candidatus_Solibacter
g__Christensenellaceae_R-7_group
g__Dongia
g__Geobacter
g__Haliangium
g__Lacunisphaera
g__Leptolinea
g__Methy locy stis
g__MND1
g__NA
g__Quadrisphaera
g__Rhizomicrobium
g__Ruminiclostridium
g__Sideroxy dans
g__Sphingomonas
g__Spirochaeta
g__Tolumonas
g__Treponema
g__UTCFX1
g__WCHB1-32
Other

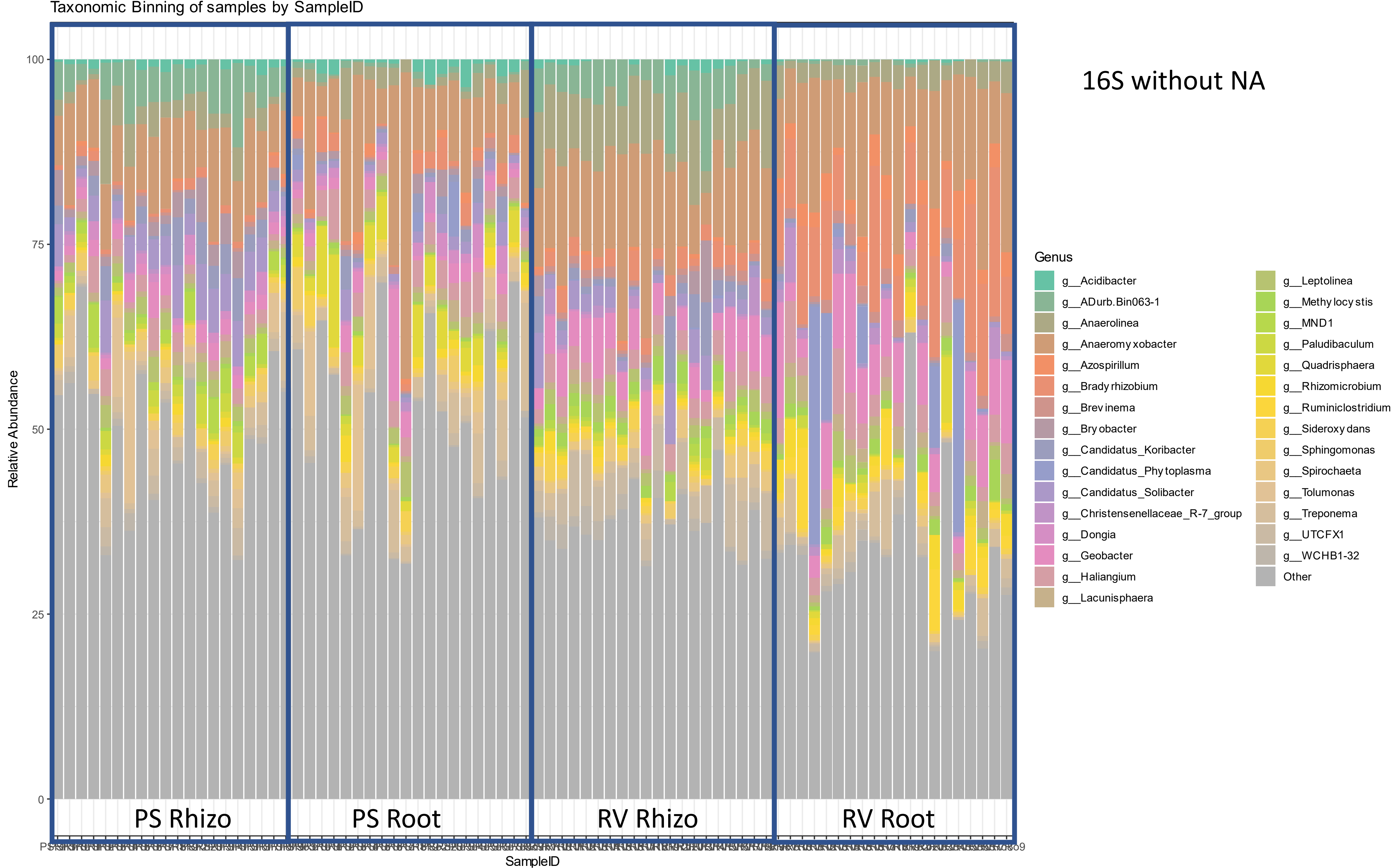
Taxonomic Binning of samples by SampleID
16S without NA
Relative Abundance
100
75
50
25
0
PS Rhizo
PS Root
RV Rhizo
RV Root
SampleID
Genus
g__Acidibacter
g__ADurb.Bin063-1
g__Anaerolinea
g__Anaeromyxobacter
g__Azospirillum
g__Bradyrhizobium
g__Brevinema
g__Bryobacter
g__Candidatus_Koribacter
g__Candidatus_Phytoplasma
g__Candidatus_Solibacter
g__Christensenellaceae_R-7_group
g__Dongia
g__Geobacter
g__Haliangium
g__Lacunisphaera
g__Leptolinea
g__Methylocystis
g__MND1
g__Paludibaculum
g__Quadrisphaera
g__Rhizomicrobium
g__Ruminiclostridium
g__Sideroxydans
g__Sphingomonas
g__Spirochaeta
g__Tolumonas
g__Treponema
g__UTCFX1
g__WCHB1-32
Other

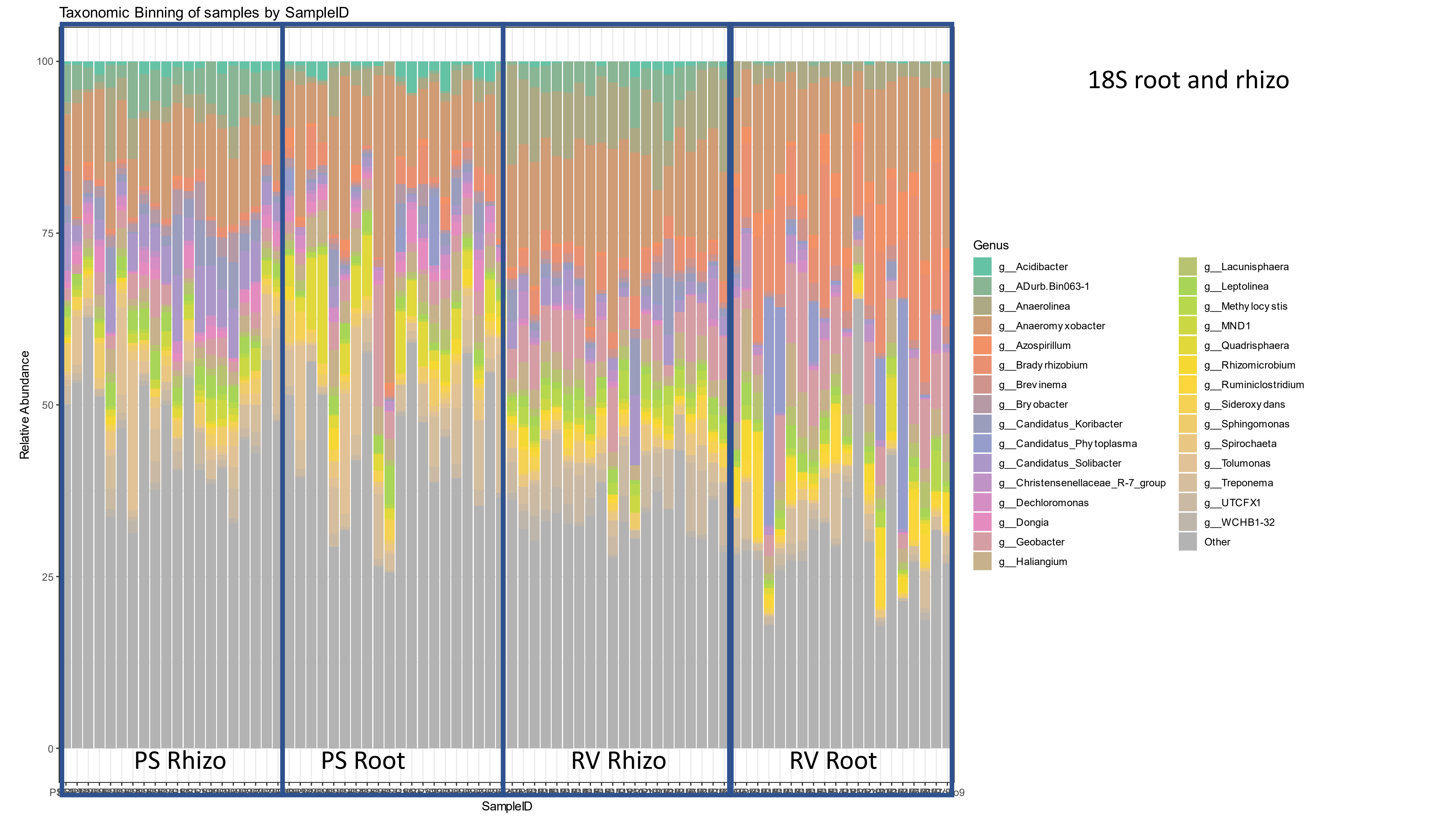

Taxonomic Binning of samples by SampleID
18S root and rhizo
Relative Abundance
100
75
50
25
0
PS Rhizo
PS Root
RV Rhizo
RV Root
SampleID
Genus
g__Acidibacter
g__ADurb.Bin063-1
g__Anaerolinea
g__Anaeromyxobacter
g__Azospirillum
g__Bradyrhizobium
g__Brevinema
g__Bryobacter
g__Candidatus_Koribacter
g__Candidatus_Phytoplasma
g__Candidatus_Solibacter
g__Christensenellaceae_R-7_group
g__Dechloromonas
g__Dongia
g__Geobacter
g__Haliangium
g__Lacunisphaera
g__Leptolinea
g__Methylocystis
g__MND1
g__Quadrisphaera
g__Rhizomicrobium
g__Ruminiclostridium
g__Sideroxydans
g__Sphingomonas
g__Spirochaeta
g__Tolumonas
g__Treponema
g__UTCFX1
g__WCHB1-32
Other

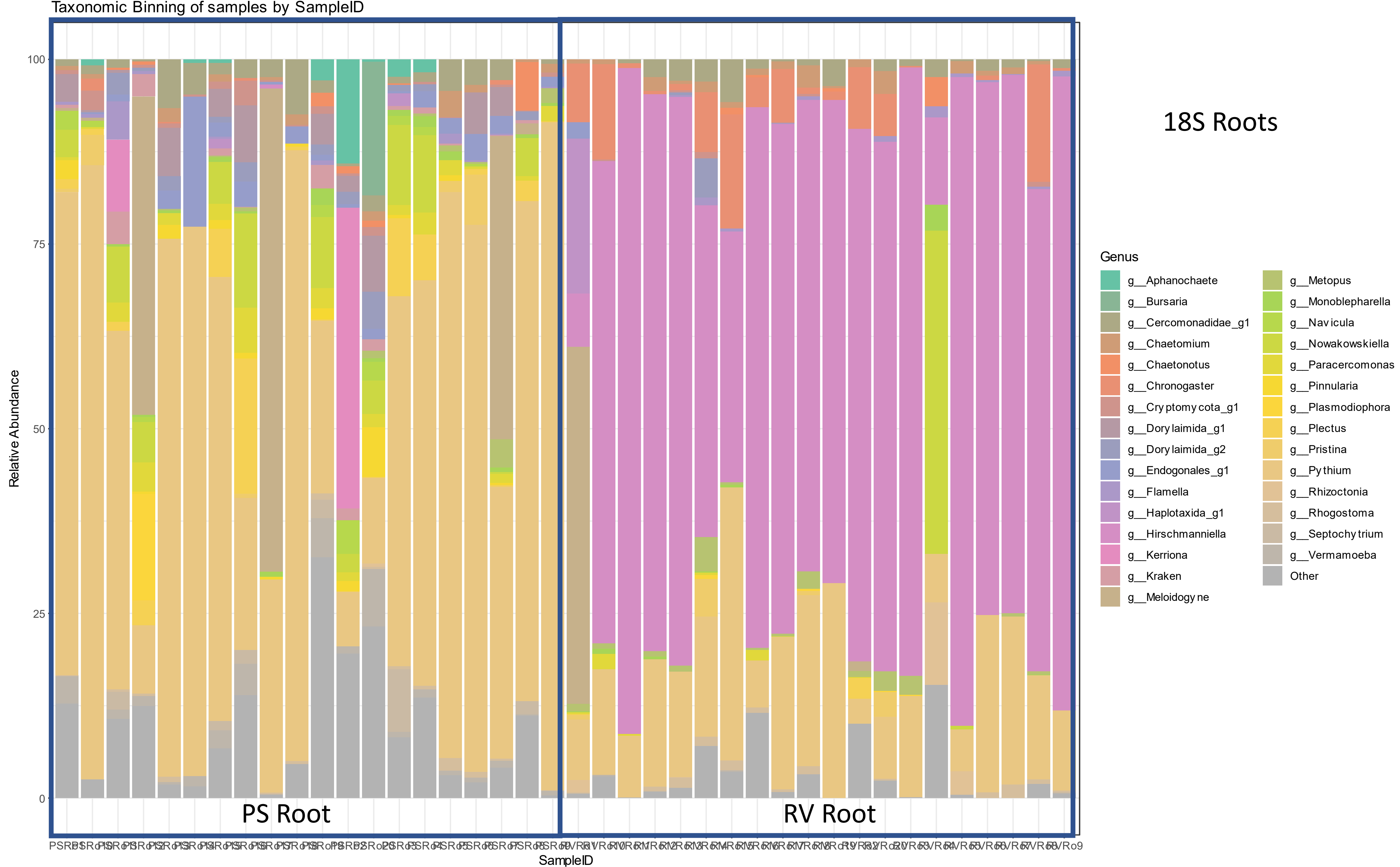
Taxonomic Binning of samples by SampleID
18S Roots
PS Root
RV Root
Relative Abundance
SampleID
0
25
50
75
100
Genus
g__Aphanochaete
g__Bursaria
g__Cercomonadidae_g1
g__Chaetomium
g__Chaetonotus
g__Chronogaster
g__Cryptomycota_g1
g__Dorylaimida_g1
g__Dorylaimida_g2
g__Endogonales_g1
g__Flamella
g__Haplotaxida_g1
g__Hirschmanniella
g__Kerriona
g__Kraken
g__Meloidogyne
g__Metopus
g__Monoblepharella
g__Navicula
g__Nowakowskiella
g__Paracercomonas
g__Pinnularia
g__Plasmodiophora
g__Plectus
g__Pristina
g__Pythium
g__Rhizoctonia
g__Rhogostoma
g__Septochytrium
g__Vermamoeba
Other
