# Supplementary material for: Comparison of plant microbiota in diseased and healthy rice reveals methylobacteria as health signatures with biocontrol capabilities
Source: Front Plant Sci. 2024 Oct 29;15:1468192. doi: 10.3389/fpls.2024.1468192 (PMC11554501; doi:10.3389/fpls.2024.1468192)
Supplement: Supplementary file 5 [file DataSheet5.pdf]

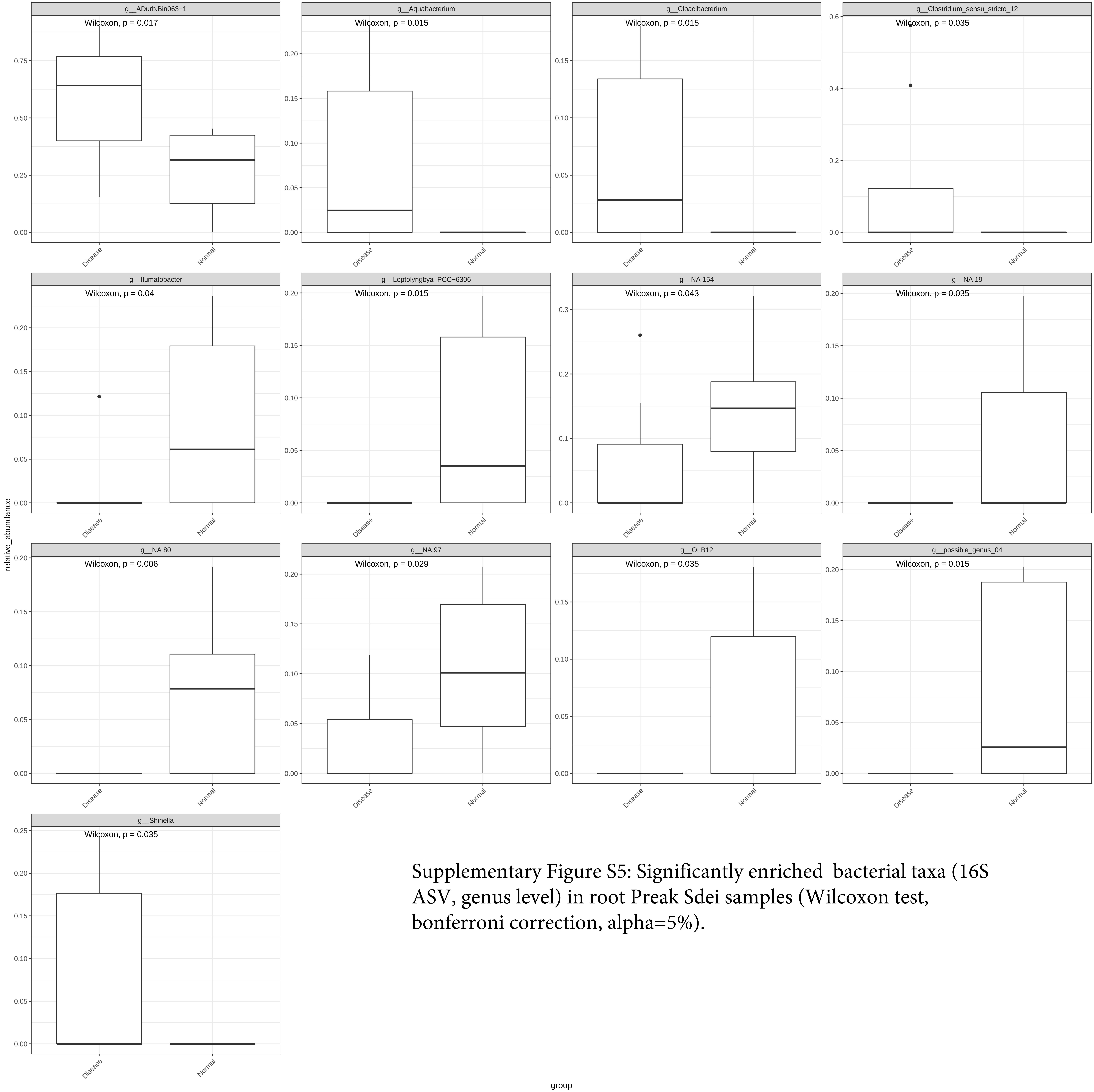

Supplementary Figure S5: Significantly enriched bacterial taxa (16S ASV, genus level) in root Preak Sdei samples (Wilcoxon test, bonferroni correction, alpha=5%).

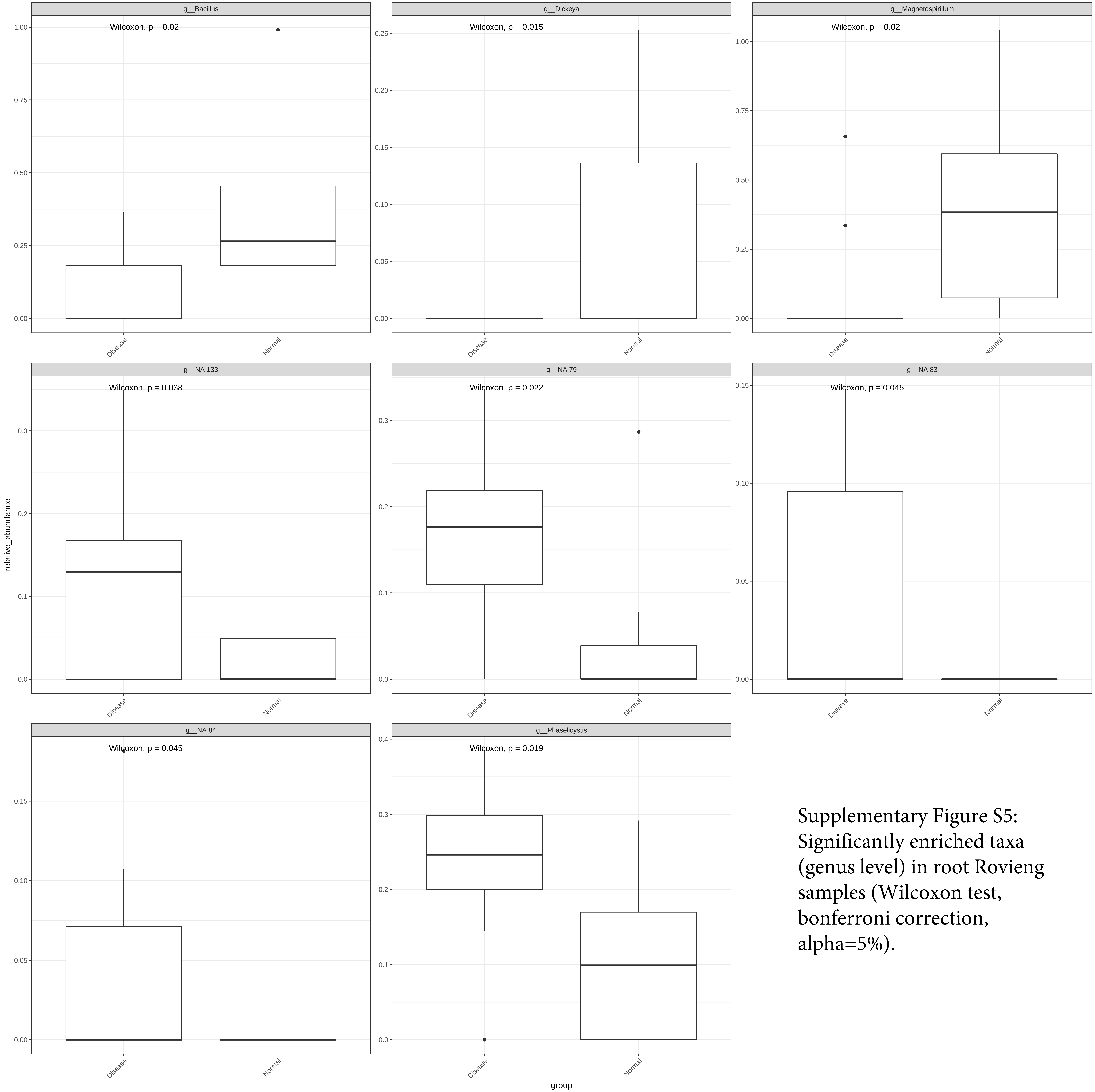

Supplementary Figure S5: Significantly enriched taxa (genus level) in root Rovieng samples (Wilcoxon test, bonferroni correction, alpha=5%).

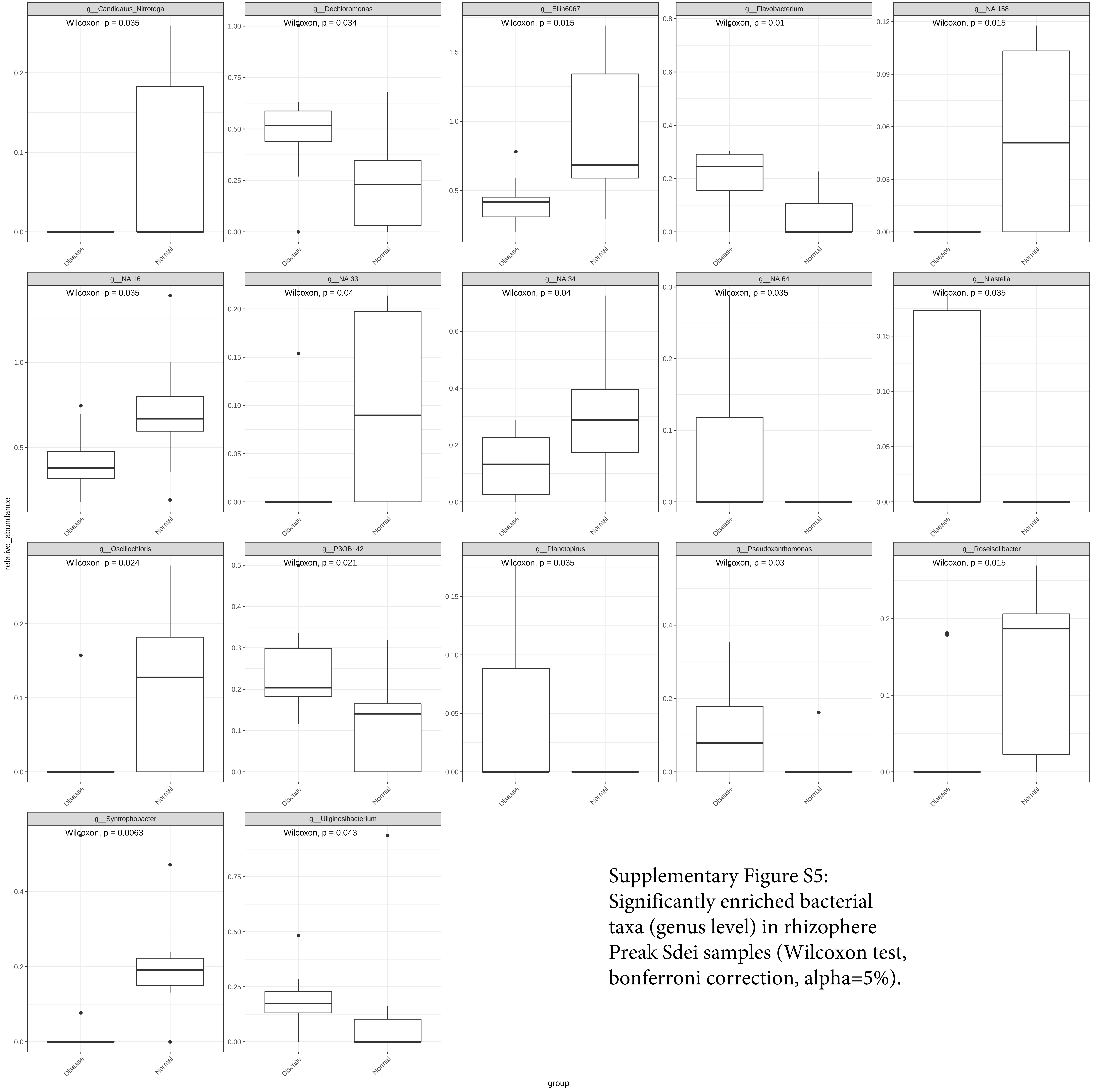

Supplementary Figure S5: Significantly enriched bacterial taxa (genus level) in rhizophere Preak Sdei samples (Wilcoxon test, bonferroni correction, alpha=5%).

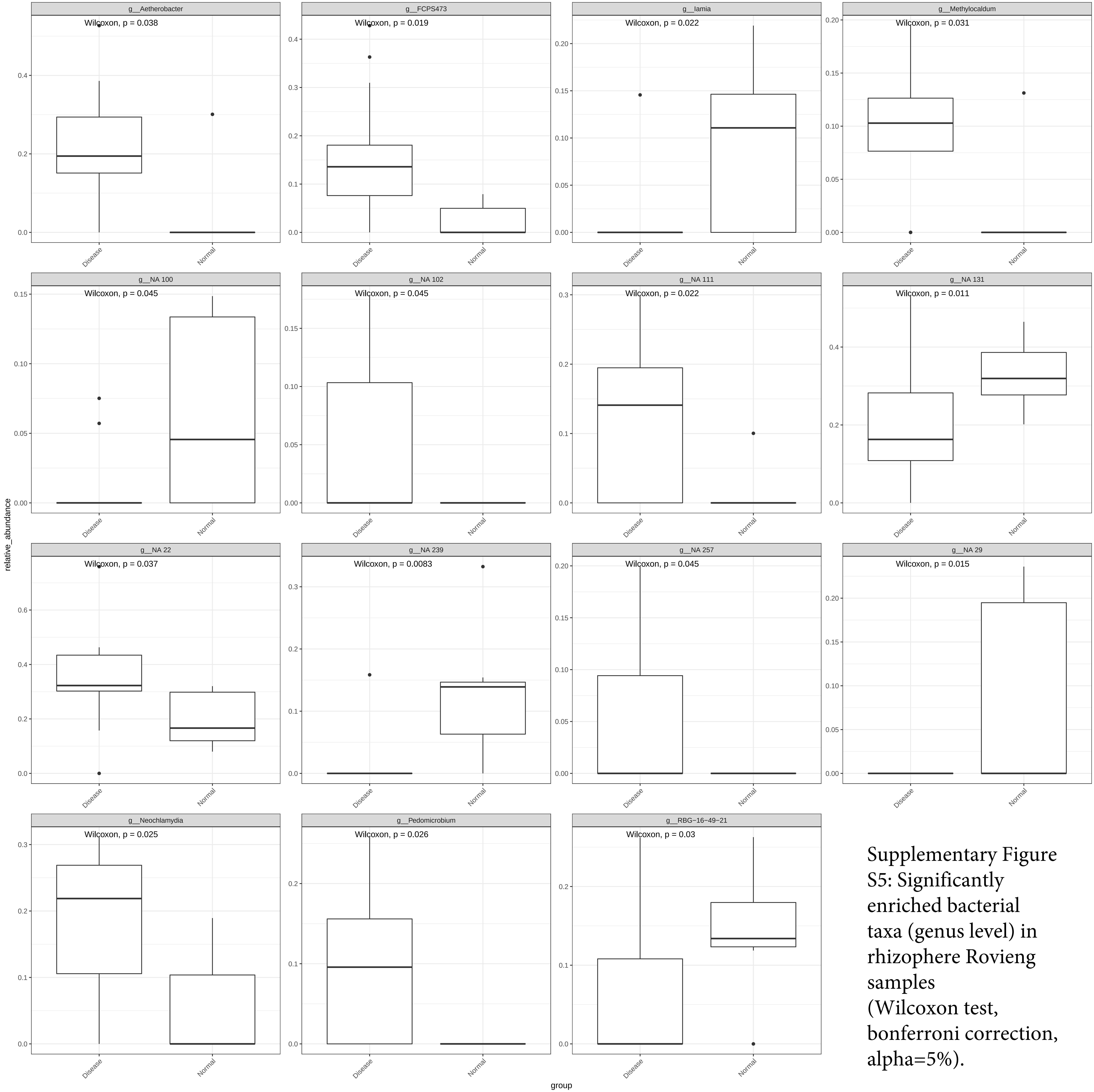

Supplementary Figure S5: Significantly enriched bacterial taxa (genus level) in rhizophere Rovieng samples (Wilcoxon test, bonferroni correction, alpha=5%).

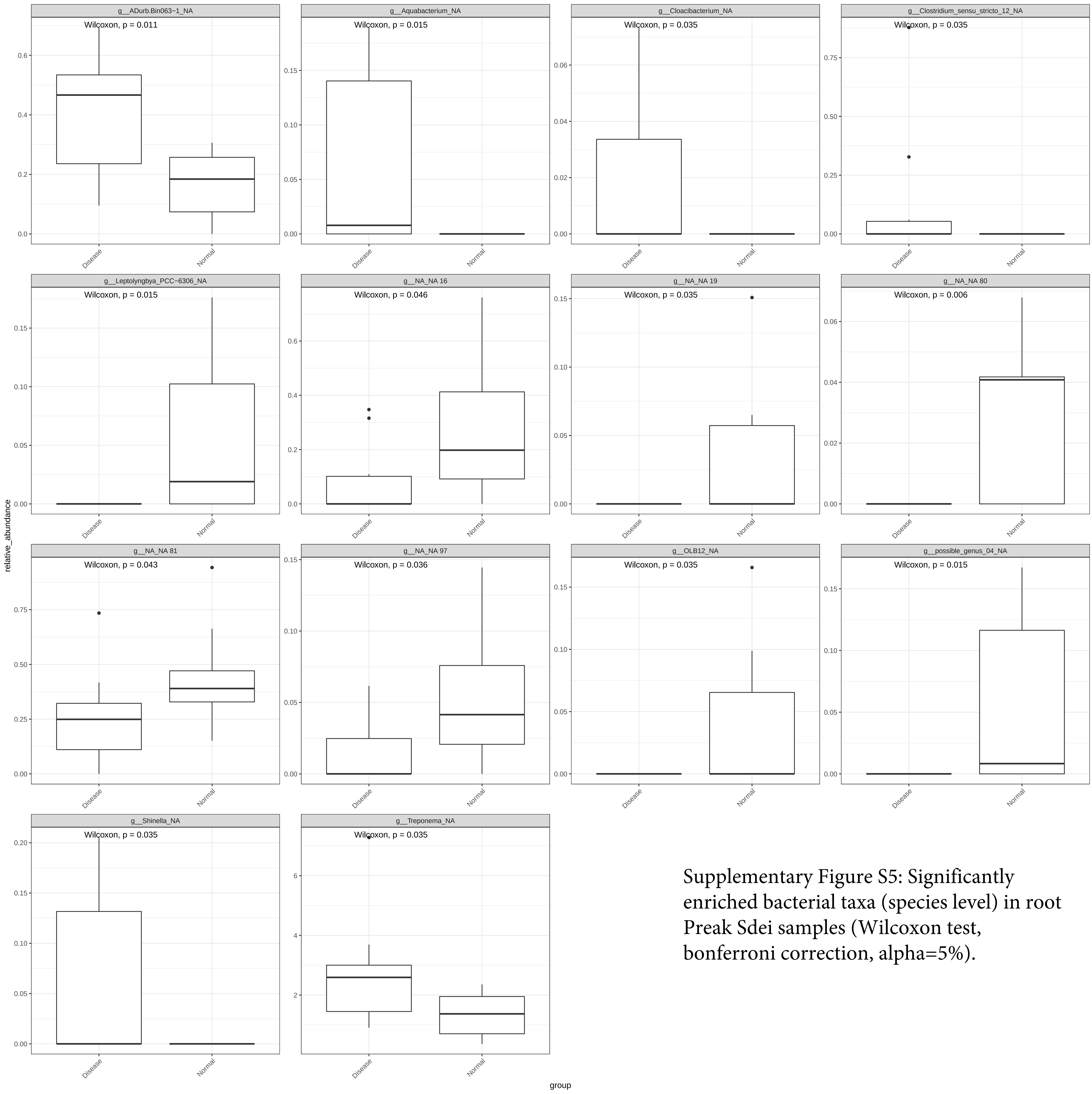

Supplementary Figure S5: Significantly enriched bacterial taxa (species level) in root Preak Sdei samples (Wilcoxon test, bonferroni correction, alpha=5%).

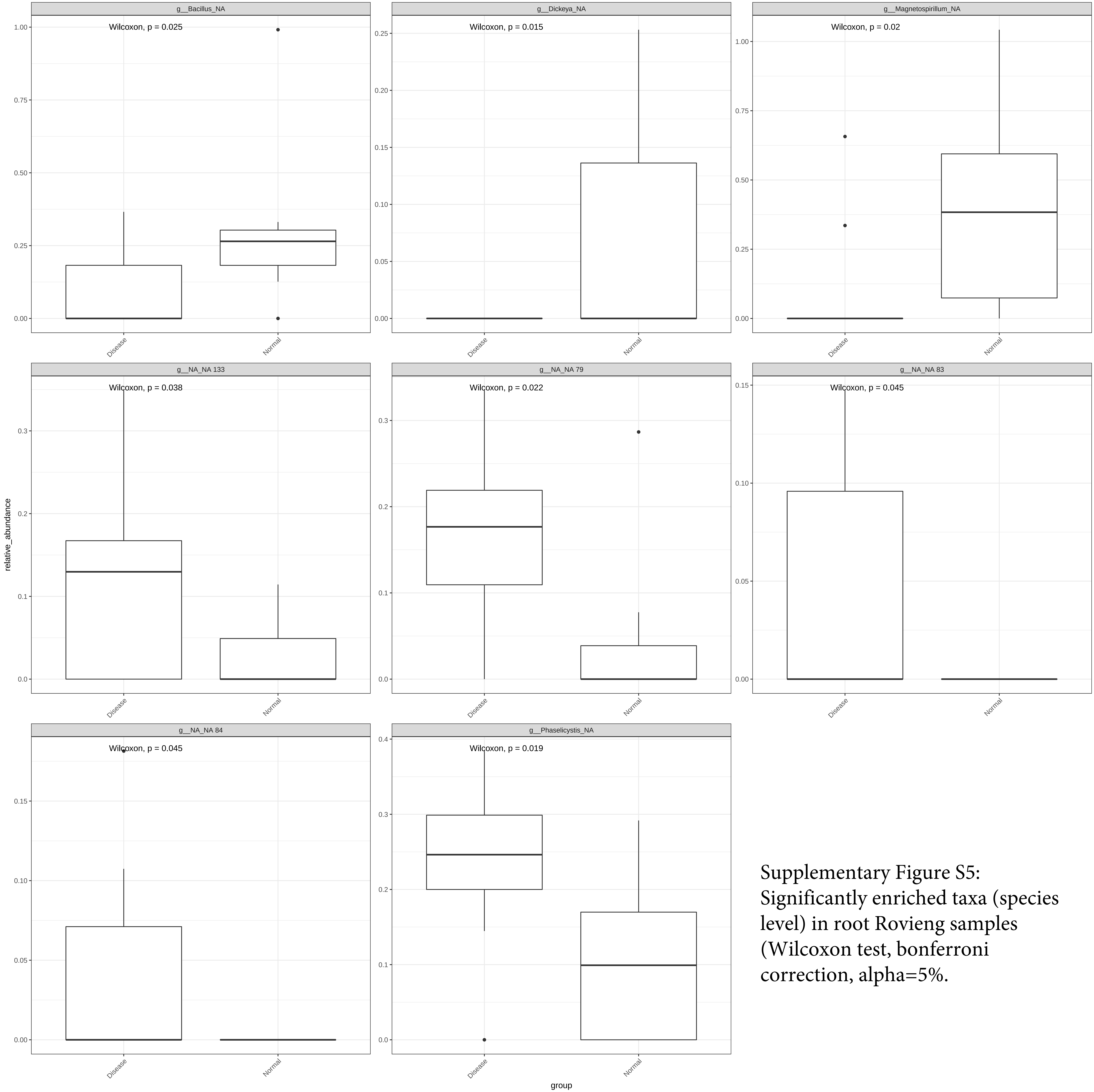

Supplementary Figure S5: Significantly enriched taxa (species level) in root Rovieng samples (Wilcoxon test, bonferroni correction, alpha=5%.

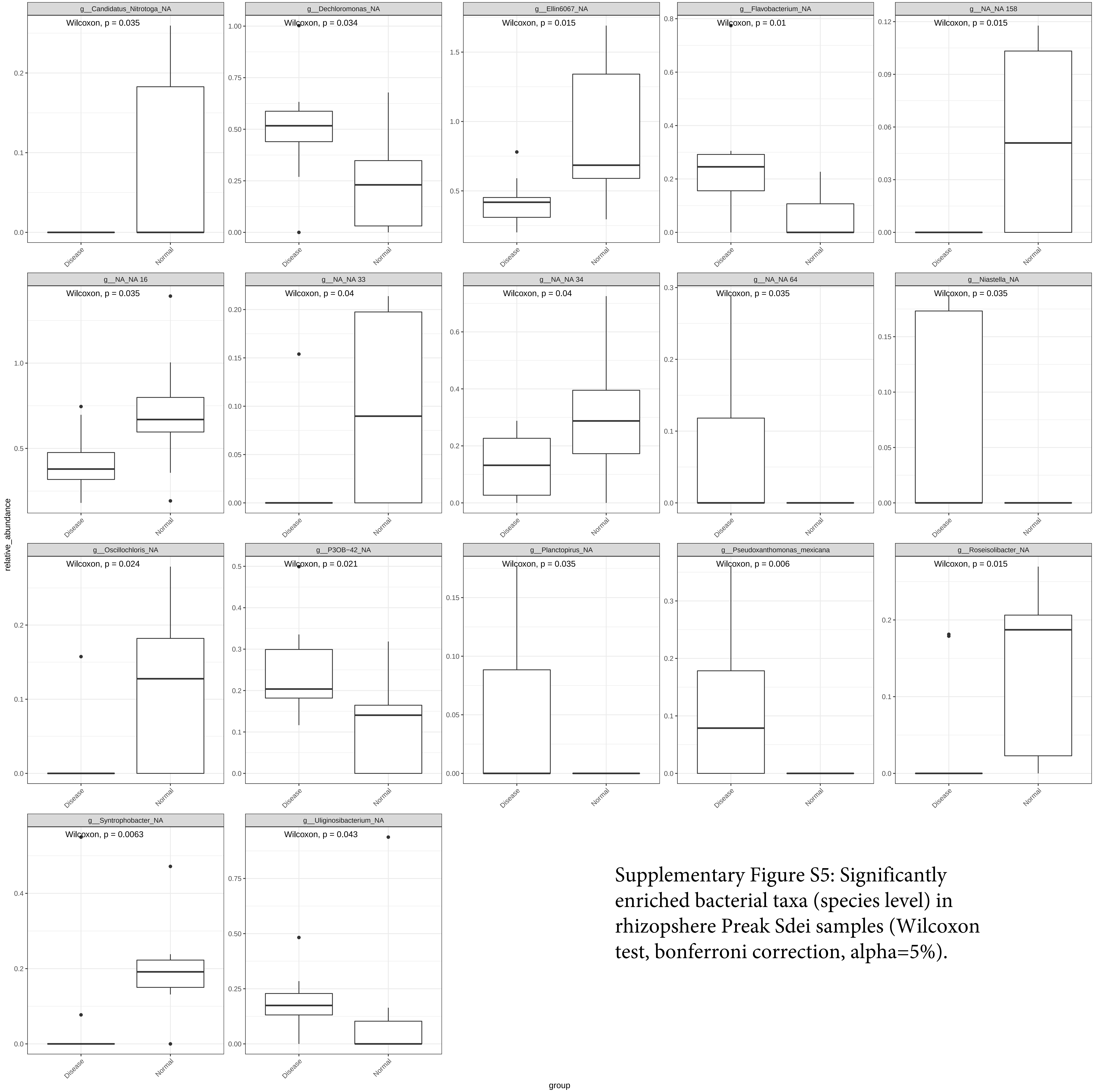

Supplementary Figure S5: Significantly enriched bacterial taxa (species level) in rhizopshere Preak Sdei samples (Wilcoxon test, bonferroni correction, alpha=5%).

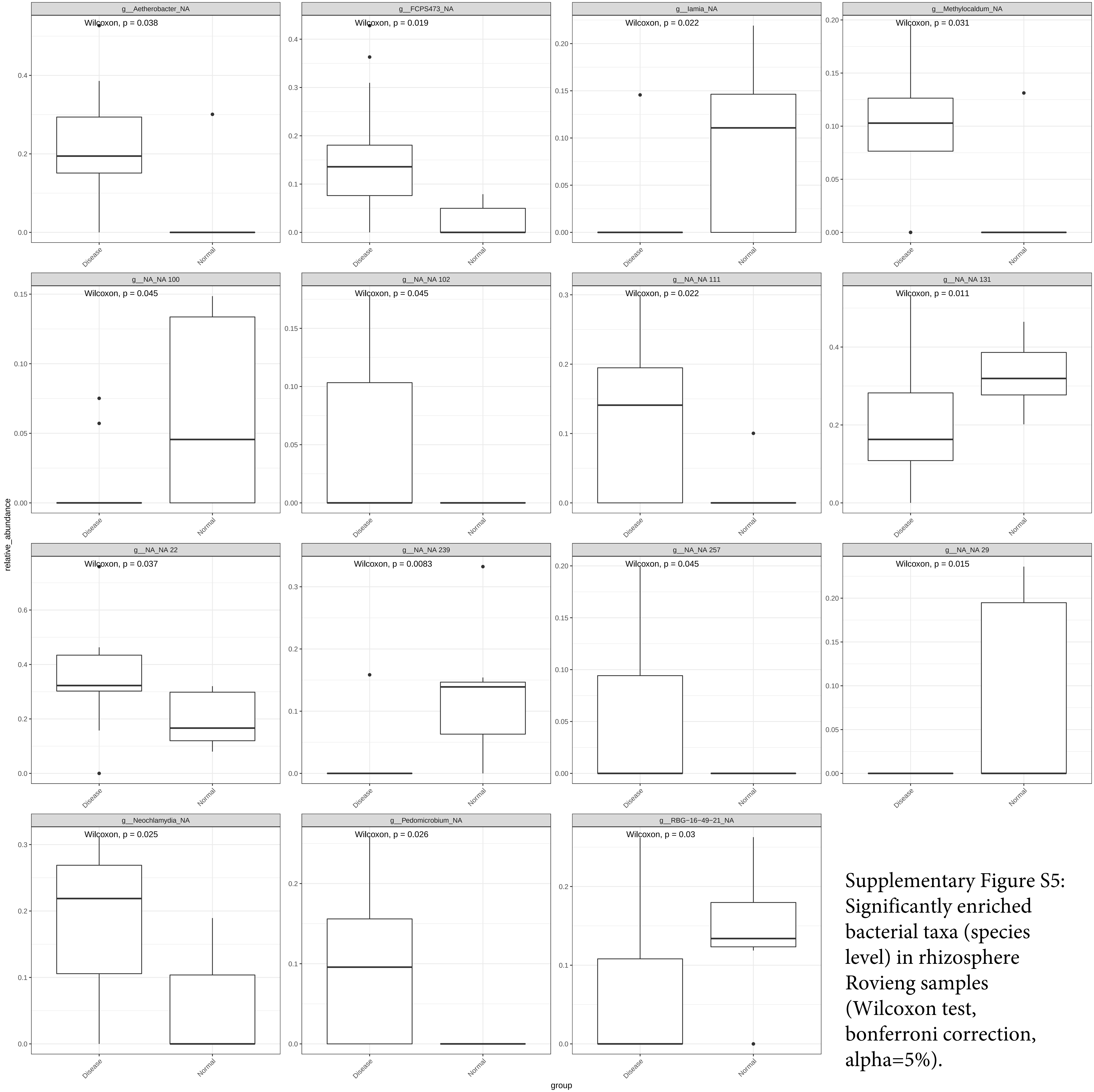

Supplementary Figure S5: Significantly enriched bacterial taxa (species level) in rhizosphere Rovieng samples (Wilcoxon test, bonferroni correction, alpha=5%).
