# Supplementary figures and images for: Comparison of plant microbiota in diseased and healthy rice reveals methylobacteria as health signatures with biocontrol capabilities
Source: Front Plant Sci. 2024 Oct 29;15:1468192. doi: 10.3389/fpls.2024.1468192 (PMC11554501; doi:10.3389/fpls.2024.1468192)

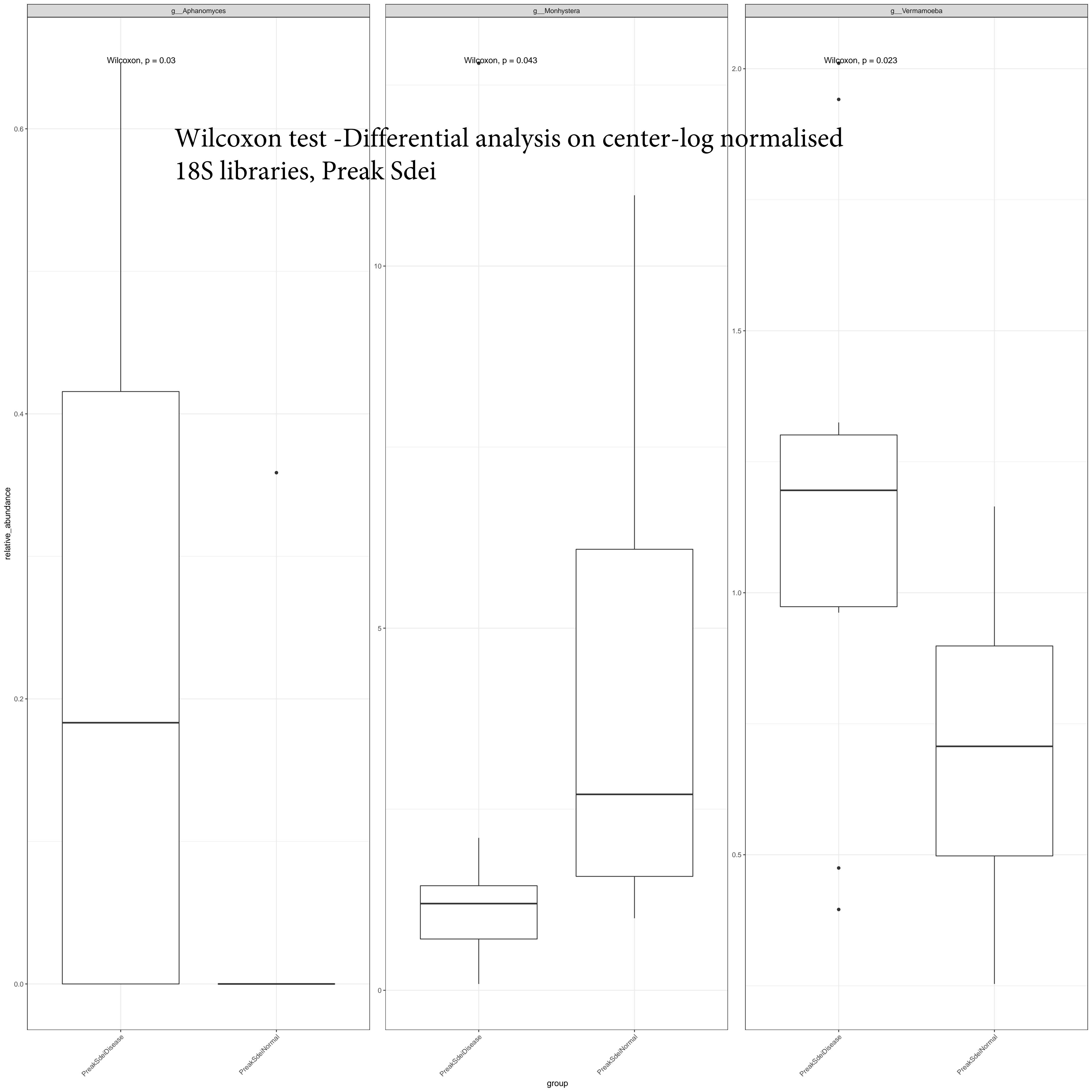

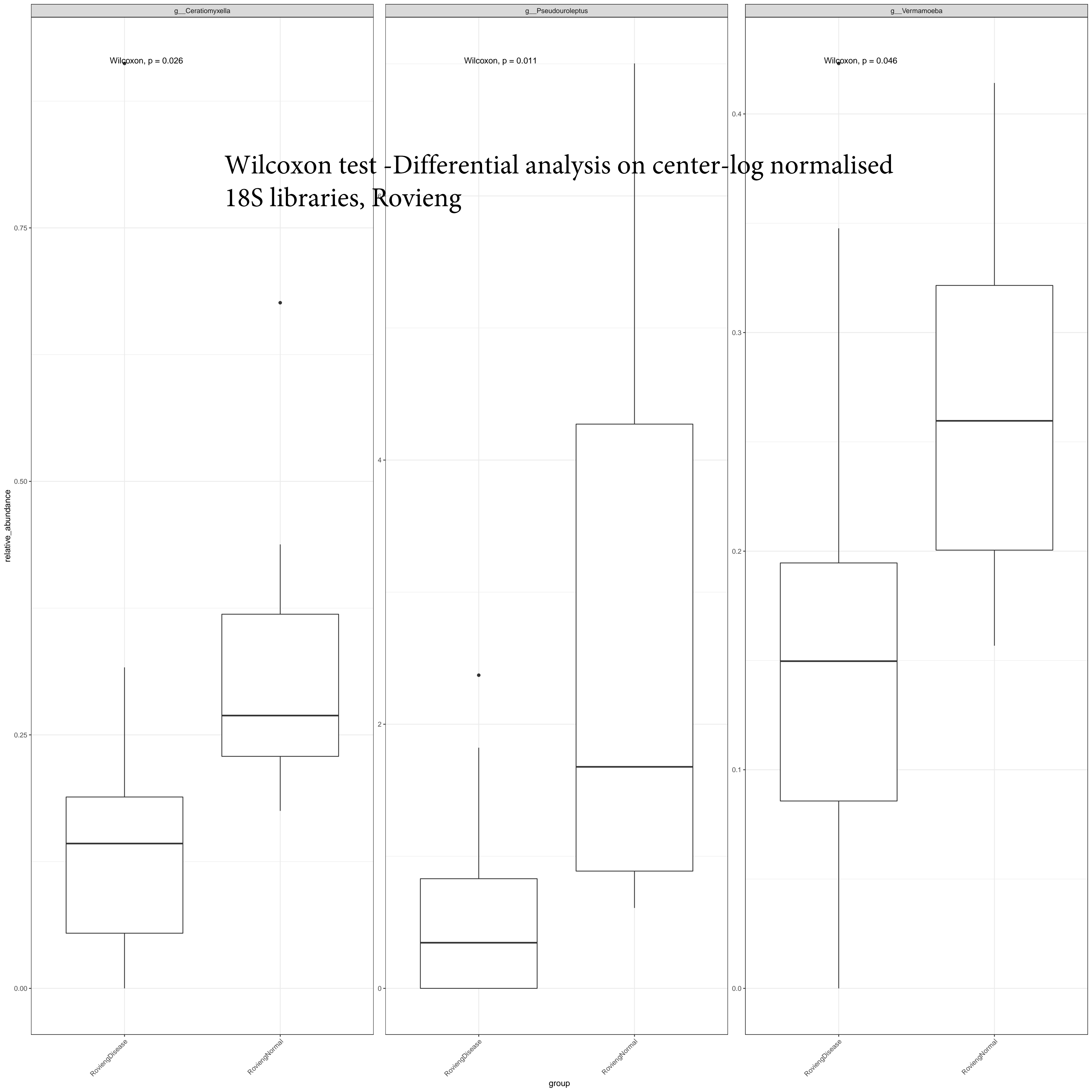

Supplement: Supplementary file 6 [file DataSheet6.pdf]
